# Genomic evidence of speciation by fusion in a recent radiation of grasshoppers

**DOI:** 10.1111/evo.14508

**Published:** 2022-06-13

**Authors:** Víctor Noguerales, Joaquín Ortego

**Affiliations:** ^1^ Department of Biological Sciences University of Cyprus Nicosia 1678 Cyprus; ^2^ Island Ecology and Evolution Group Instituto de Productos Naturales y Agrobiología (IPNA‐CSIC) San Cristóbal de La Laguna 38206 Spain; ^3^ Department of Integrative Ecology Estación Biológica de Doñana (EBD‐CSIC) Sevilla 41092 Spain

**Keywords:** Demographic modeling, hybrid speciation, hybridization, introgression, trophic niche expansion

## Abstract

Postdivergence gene flow can trigger a number of creative evolutionary outcomes, ranging from the transfer of beneficial alleles across species boundaries (i.e., adaptive introgression) to the formation of new species (i.e., hybrid speciation). Although neutral and adaptive introgression has been broadly documented in nature, hybrid speciation is assumed to be rare and the evolutionary and ecological context facilitating this phenomenon still remains controversial. Through combining genomic and phenotypic data, we evaluate the hypothesis that the dual feeding regime (based on both scrub legumes and gramineous herbs) of the taxonomically controversial grasshopper *Chorthippus saulcyi algoaldensis* resulted from hybridization between the sister taxa *C. binotatus* (that exclusively feeds on scrub legumes) and *C. saulcyi* (that only feeds on gramineous herbs). Genetic clustering analyses and inferences from coalescent‐based demographic simulations confirm that *C. s. algoaldensis* represents an independently evolving lineage and support the ancient hybrid origin of this taxon (about 1.4 Ma), which sheds light on its uncertain phylogenetic position and might explain its broader trophic niche. We propose a Pleistocene hybrid speciation model where range shifts resulting from climatic oscillations can promote the formation of hybrid swarms and facilitate their long‐term persistence through geographic isolation from parental forms in topographically complex landscapes.

Gene flow is recognized as a fundamental control on the speciation process, with disparate evolutionary consequences that can impact either positively or negatively the formation and persistence of independently evolving lineages (Mallet 2007; Dynesius and Jansson 2014). At one extreme, gene flow can inhibit the onset of speciation by homogenizing gene pools (Slatkin 1985; Dynesius and Jansson 2014) and lead to “speciation reversal” if lineages that have remained isolated for extended periods of time merge back into one after secondary contact (Seehausen et al. 2008; Kleindorfer et al. 2014; Kearns et al. 2018). At the opposite extreme, gene flow can generate a wide spectrum of creative evolutionary outcomes, ranging from adaptive introgression across species boundaries (Hedrick 2013; Suarez‐Gonzalez et al. 2018a) to the formation of new hybrid species (Mallet 2007). Introgressive hybridization as a source of novel alleles conferring advantages to the recipient species has been widely documented in numerous organism groups (Hedrick 2013; Suarez‐Gonzalez et al. 2018b). This phenomenon can lead to the acquisition of new traits, including the capacity to exploit new host plants (Aardema and Andolfatto 2016), Müllerian mimicry (Pardo‐Diaz et al. 2012; Enciso‐Romero et al. 2017), and resistance to herbivores (Whitney et al. 2006), and has been proven to be instrumental in niche expansions (Scascitelli et al. 2010; Malinsky et al. 2018) and adaptation to suboptimal environmental conditions (Pfennig et al. 2016; Suarez‐Gonzalez et al. 2018a; Leroy et al. 2020). In other cases, hybridization promotes the formation of new species (i.e., hybrid speciation) through the emergence of evolutionary innovations and reproductive isolation between parental and hybrid lineages (Gross and Rieseberg 2005; e.g., Gompert et al. 2006; Nice et al. 2013). Beyond a sporadic and fortuitous phenomenon, hybridization and introgression have been hypothesized to be responsible of fueling (“Syngameon” hypothesis; Seehausen 2004; Seehausen et al. 2014; e.g., Patton et al. 2020) or even igniting the onset of adaptive radiations (“Hybrid swarm origin of adaptive radiation” hypothesis; Meier et al. 2017).

Although introgression is rampant across the Tree of Life and allopolyploid hybrid speciation is relatively frequent in plants, homoploid hybrid speciation—speciation via hybridization without a change in chromosome number—has been much more rarely documented (Mallet 2007; Schumer et al. 2014; Taylor and Larson 2019). The major challenge for homoploid hybrid species to persist through evolutionary time is eluding the homogenizing effects of gene flow with their sympatric progenitors. Despite its evolutionary significance, the specific evolutionary and ecological contexts facilitating hybrid speciation are still controversial from both a theoretical and empirical perspective (Buerkle et al. 2000; Servedio et al. 2013; Schumer et al. 2014). What is well understood is that hybridization should have direct consequences on fitness of the incipient hybrid species through the emergence of novel or intermediate phenotypes on which natural or sexual selection can act on (Mavárez et al. 2006; Hedrick 2013; Taylor and Larson 2019). Literature on homoploid hybrid speciation has linked this phenomenon to rapid reproductive isolation between parental and hybrid lineages through strong assortative mating (Mavárez et al. 2006; Melo et al. 2009), colonization and adaptation to novel habitats with extreme conditions (Rieseberg et al. 1996, Rieseberg et al. 2003; Gompert et al. 2006), or exploitation of new trophic resources (Schwarz et al. 2005; Lamichhaney et al. 2018). However, successful homoploid hybrid speciation generally implies the co‐occurrence of ecological circumstances (e.g., opening and colonization of a new niche space unavailable to either parental species), genetic mechanisms (e.g., chromosomal rearrangements, etc.), and phenotypic changes (e.g., recombinant or transgressive morphologies) that increase the capacity to exploit novel resources and promote reproductive isolation (McCarthy et al. 1995; Gross and Rieseberg 2005; Schumer et al. 2014; Lamichhaney et al. 2018).

The species group *Chorthippus* (*Glyptobothrus*) (*binotatus*) (Charpentier, 1825) is a recently diverged complex of grasshoppers distributed in the westernmost portion of the Palearctic (Cigliano et al. 2021; Figs. 1 and 2). The complex is composed of eight taxa grouped in two major clades—*C. binotatus* and *C. saulcyi* clades—that exhibit distinct host‐plant associations (Fig. 1; Defaut 2011; Noguerales et al. 2018a). Although taxa from the clade *C. binotatus* exclusively feed on scrub legumes (Fabaceae, tribe Genisteae), lineages within the clade *C. saulcyi* show a feeding regime based on gramineous herbs (Poaceae; Picaud et al. 2003; Defaut 2011). The only exception is *C. saulcyi algoaldensis*, a narrow‐endemic taxon distributed in the Massif Central (France; Fig. 2) that feeds on both scrub legumes and gramineous herbs (Fig. 1; Defaut 2011; Noguerales et al. 2018a). This taxon also presents a distinctive male calling song structure and an intermediate morphological position between its putative (*C. saulcyi*) and sister (*C. binotatus*) clades (Defaut 2011). Although a preliminary study strongly supported the distinctiveness of each taxon within the complex, the phylogenetic placement of *C. s. algoaldensis* as a sister lineage to the rest of taxa within either *C. saulcyi* or *C. binotatus* clades remained unresolved (Noguerales et al. 2018a). Collectively, all these pieces of evidence raise the hypothesis of introgressive hybridization or speciation by fusion as a potential explanation for the broader trophic niche of *C. s. algoaldensis* and its uncertain phylogenetic position, an evolutionary history departing from expectations under a strictly bifurcating model of divergence that should have left a distinctive signature on its genome (Meng and Kubatko 2009). Given that the rapid history of diversification of the complex is likely explained by processes of allopatric speciation during the Pleistocene (Mayer et al. 2010; Noguerales et al. 2018a, 2018b), distributional shifts driven by climatic fluctuations could have also provided ample opportunities for secondary contact and admixture among recently diverged lineages in which reproductive barriers to gene flow might be incomplete or absent (Hewitt 1999; Nolen et al. 2020; Ortego and Knowles 2021).

**Figure 1:**
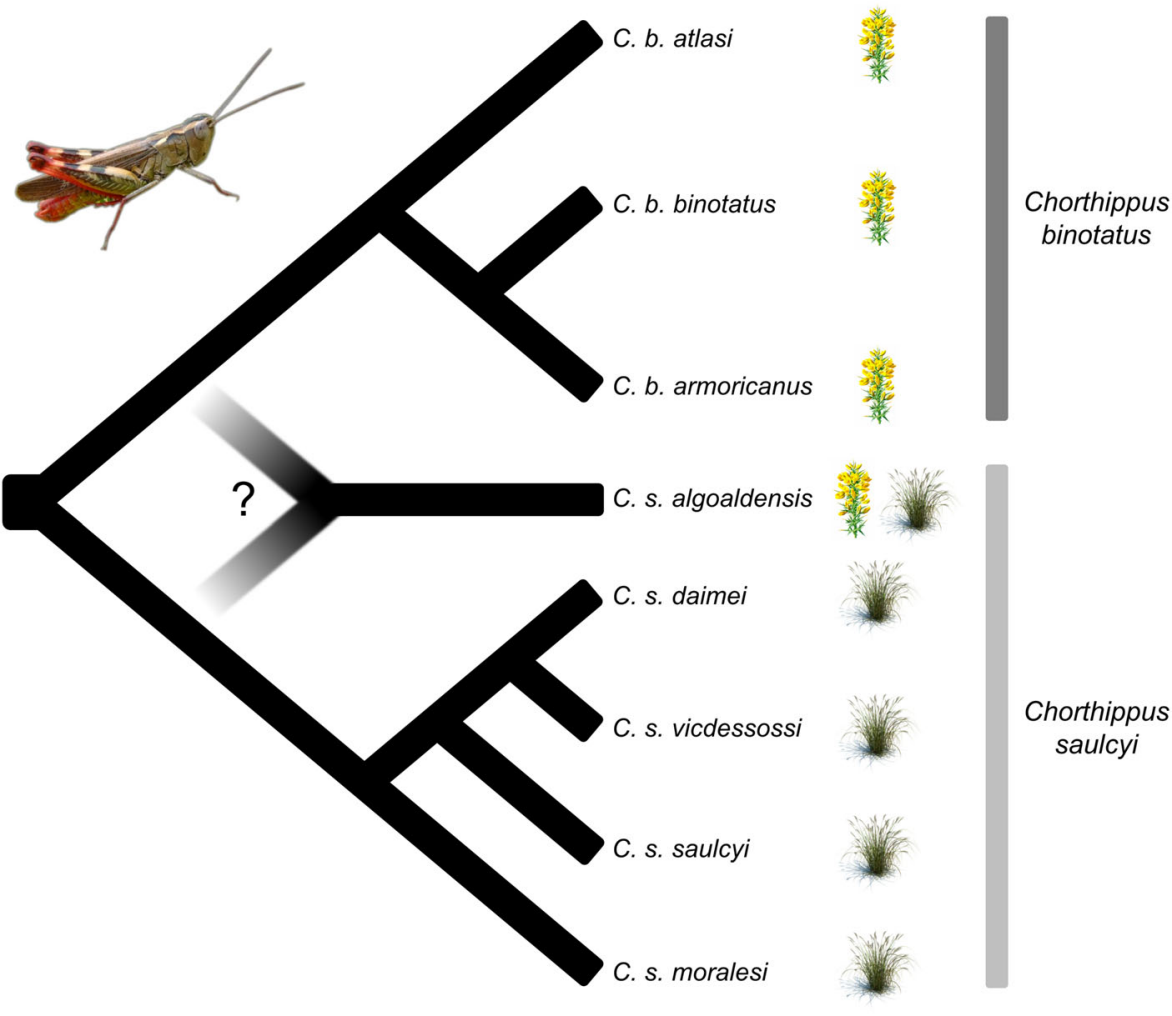
Schematic showing the phylogenetic relationships and host‐plant associations (scrub legumes vs. gramineous herbs) for the different taxa within the studied species complex and the taxonomic and phylogenetic uncertainties around the focal taxon *C. s. algoaldensis*. In this study, we test alternative hypotheses concerning the roles of introgression and hybridization in the evolutionary history of *C. s. algoaldensis*, which has been traditionally included within *C. saulcyi* according to its morphology but shows dual host‐plant associations and presents an uncertain phylogenetic placement (Noguerales et al. 2018a).

**Figure 2:**
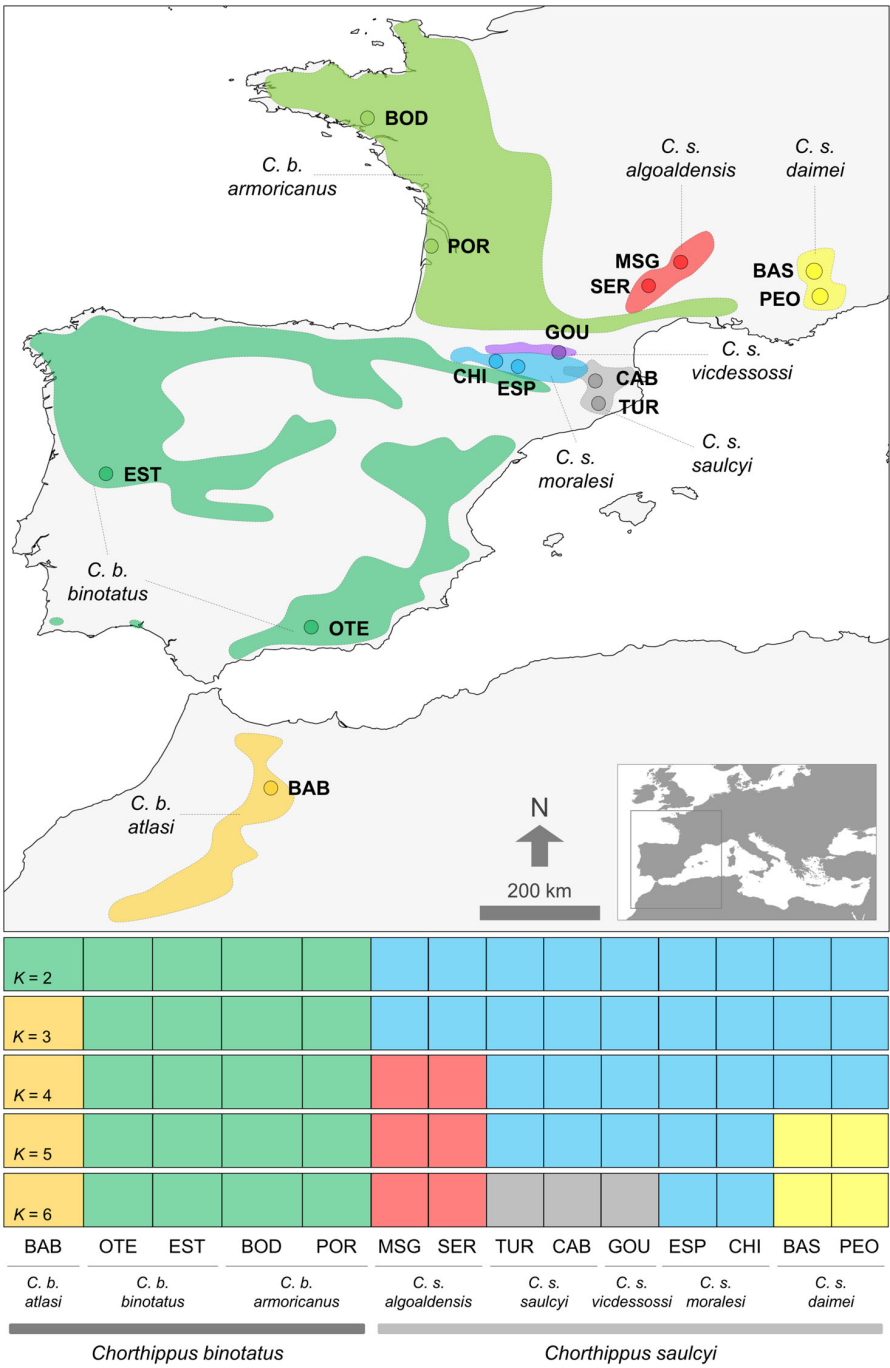
Geographical location of the sampled populations and distribution range of the different taxa from the studied species complex. Panels on the bottom show inferred genetic clustering from *K* = 2 to *K* = 6, the best‐supported solutions as inferred by faststructure. Individuals are partitioned into *K* colored segments representing the probability of belonging to the cluster with that color. Thin vertical black lines separate different populations. Population codes as in Table S1.

In this study, we integrate genomic data obtained through double‐digest restriction site‐associated DNA sequencing (ddRADseq) and phenotypic information to test the hypothesis that *C. s. algoaldensis*, which feeds on both scrub legumes and gramineous herbs, resulted from hybridization between parental lineages with narrower host‐plant requirements. Leveraging inferences obtained through both phylogenomics and population genetics frameworks, we evaluate the specific pathways that might have hypothetically led to the evolution of the contrasting feeding strategies within the studied species complex. First, we perform clustering analyses to determine the genetic distinctiveness and cohesiveness of putative taxa within the complex and detect signatures of ongoing hybridization or recent admixture across species boundaries. Second, we test whether the unresolved phylogenetic position of *C. s. algoaldensis* is explained by incomplete lineage sorting or introgression. Third, we use a model‐based simulation approach to test refined hypotheses invoking alternative scenarios of diversification, namely, strict bifurcation, introgression, and speciation by fusion, and infer the mode and estimate the timing of species formation. Finally, we test whether genomic‐based inferences are congruent with phenotypic variation at different morphometric traits of taxonomic value in the group (Noguerales et al. 2018a), predicting either intermediate or transgressive phenotypes in *C. s. algoaldensis* if introgression and speciation by fusion are supported as the most likely scenarios explaining the origin of this taxon (Rieseberg et al. 1999; Rheindt et al. 2014; Thom et al. 2018).

## Materials and Methods

### SAMPLE COLLECTION

We collected samples from the eight putative taxa (14 populations, 231 individuals) constituting the species group *Chorthippus* (*Glyptobothrus*) *binotatus* (Charpentier 1825) (Noguerales et al. 2018a and references therein; Table S1; Figs. 1 and 2). When possible, we collected two populations per taxon and tried to maximize the distance from each other to include samples representative of their respective distribution ranges (Table S1; Fig. 2). We used all collected individuals for morphometric analyses (*n* = 231 specimens, 16–20 individuals per population) and a subset of them for genomic analyses (*n* = 77 specimens, 5–7 individuals per population; Table S1).

### GENOMIC DATA

We complemented the genomic dataset from Noguerales et al. (2018a) with the addition of 37 newly genotyped individuals, including those from seven new populations (Table S1). We extracted DNA from a total of 77 individuals (Table S1) and processed them into two genomic libraries following the ddRADseq procedure described in Peterson et al. (2012). We also included into the libraries six individuals from *Chorthippus* (*Glyptobothrus*) *biroi* (Kuthy 1907) (Table S1), which were used as an outgroup in phylogenomic and introgression/hybridization analyses. Details on the preparation of ddRADseq libraries are presented in Methods S1. Raw sequences were demultiplexed and preprocessed using stacks version 1.35 (Catchen et al. 2013) and assembled in pyrad version 3.0.66 (Eaton 2014). All downstream analyses are based on datasets of unlinked SNPs (i.e., one SNP per locus). Methods S2 provides all details on data filtering and sequence assembling. We used the option *relatedness2* in vcftools to calculate the relatedness between all pairs of genotyped individuals and exclude the possibility that we had sampled close relatives (Manichaikul et al. 2010; Danecek et al. 2011).

### GENETIC CLUSTERING ANALYSES

Genetic clustering of the studied taxa and populations was inferred using the variational Bayesian framework implemented in faststructure version 1.0 (Raj et al. 2014). Ten independent replicates were performed for a range of different *K* genetic clusters (*K* = 1–14) using a flat beta‐prior over population‐specific allele frequencies at each locus (“simple” prior) and a convergence criterion of 1 × 10^−7^ (Raj et al. 2014). The number of genetic clusters that best describes our data was assessed by calculating the metrics *K* ^*^_ø_^c^, the value of *K* that maximizes log‐marginal likelihood lower bound of the data, and *K* ^*^_ɛ_, the smallest number of model components explaining at least 99% of cumulative ancestry contribution in our sample (Raj et al. 2014). Complementarily, genetic clustering was also analyzed using a Discriminant Analysis of Principal Components (DAPC; Jombart et al. 2010). Unlike faststructure or akin methods, DAPC does not lay on model‐based assumptions and it has been suggested that DPAC could exhibit higher performance to reveal complex patterns of genetic structure (Jombart et al. 2010). The optimal number of principal components (PCs) and the best‐supported number of genetic clusters were estimated as detailed in Noguerales et al. (2016). We ran DAPC using the *adegenet* package (Jombart 2008) in r version 4.0.3 (R Core Team 2021).

### PHYLOGENOMIC INFERENCE

Phylogenomic relationships among taxa were reconstructed using matrices of unlinked SNPs and two different coalescent‐based methods for species tree estimation. First, we ran svdquartets (Chifman and Kubatko 2014) including *C. biroi* as an outgroup, exhaustively evaluating all possible quartets, and performing nonparametric bootstrapping with 100 replicates for quantifying uncertainty in relationships. Second, we used the Bayesian coalescent model implemented in snapp version 1.3 (Bryant et al. 2012). We used the *phrynomics* r package (B. Banbury, http://github.com/bbanbury/phrynomics) to remove non‐biallelic SNPs, code heterozygotes, and format the input file for snapp. The resulting dataset included 2926 biallelic unlinked SNPs shared across tips. We applied two alternative gamma distributions for the ancestral population size parameter (θ), namely G(2, 200) and G(2, 2000), and left default settings for all other parameters. We ran two independent runs for each gamma distribution using different starting seeds for ≥1.5 million Markov chain Monte Carlo (MCMC) generations, sampling every 1000 steps. Due to high computational burden of snapp analyses, the number of taxa partitions was limited by including only one population per taxon. We used tracer version 1.4 to examine log files and check stationarity and convergence of the chains and confirm that effective sample sizes (ESS) for all parameters were >200. We removed 10% of trees as burn‐in and combined tree and log files for replicated runs using logcombiner version 2.4.7. Maximum credibility trees were obtained using treeannotator version 2.4.7 and the full set of likely species trees was displayed with densitree version 2.2.6, which is expected to show fuzziness in parts of the tree due to gene flow or other causes of phylogenetic conflict (Bouckaert 2010).

### TESTING FOR INTROGRESSION

We used four‐taxon ABBA/BABA tests based on the *D*‐statistic to determine the role of hybridization/introgression in explaining unresolved phylogenetic relationships involving *C. s. algoaldensis* (Durand et al. 2011). This method enables evaluating to what extent gene‐tree incongruences have resulted from either gene flow between non‐sister taxa or retention of ancestral genetic variation (i.e., incomplete lineage sorting). Assuming that the sister species P_1_ and P_2_ diverged from P_3_ and an outgroup species O, the *D*‐statistic is used to test the null hypothesis of no introgression (*D* = 0) between P_3_ and P_1_ or P_2_. *D*‐values significantly different from 0 indicate gene flow between P_1_ and P_3_ (*D* < 0) or between P_2_ and P_3_ (*D* > 0). We assigned taxa within *C*. *saulcyi* (excluding *C. s. algoaldensis*) to P_1_, *C. s. algoaldensis* to P_2_, taxa within *C*. *binotatus* to P_3_, and *C. biroi* to the outgroup (O). We performed ABBA/BABA tests in pyrad and used 1000 bootstrap replicates to obtain the standard deviation of the *D*‐statistic (Eaton and Ree 2013). We ran ABBA/BABA tests combining data from all taxa and populations in each group (P_1_, P_2_, and P_3_) and also performing independent analyses considering each taxon independently (i.e., subspecies within *C. binotatus* and *C. saulcyi*; Table S2). To investigate introgression signatures between other pairs of nonsister subspecies from the *C*. *saulcyi* and *C*. *binotatus* clades, we also performed ABBA/BABA tests considering all other possible taxa combinations not involving the focal lineage *C. s. algoaldensis* (Table S2).

### TESTING ALTERNATIVE DEMOGRAPHIC MODELS

We evaluated alternative models of speciation for *C. s. algoaldensis* to determine whether its uncertain phylogenetic position when assuming a strictly bifurcating tree is a consequence of an introgression event from *C. binotatus* into *C. s. algoaldensis* (i.e., a pulse of gene flow) after the latter diverged from *C. saulcyi* (hereafter, “introgression model”) or if, alternatively, *C. s. algoaldensis* originated from an admixture event between *C. binotatus* and *C. saulcyi* (hereafter, “speciation by fusion model”; sensu Grant and Grant 2018; see also Barrera‐Guzman et al. 2018). Following Meier et al. (2016), these two alternative models, together with a null model considering a strictly bifurcating history of divergence (hereafter, “strictly bifurcating model”), were built assuming no migration among demes and also considering ancestral and contemporary postdivergence gene flow (i.e., a total of 12 models, illustrated in Figs. 4 and S1). To evaluate the relative statistical support for each of these alternative demographic scenarios, we estimated the composite likelihood of the observed data given a specified model using the site frequency spectrum (SFS) and the simulation‐based approach implemented in fastsimcoal2 version 2.5.2.21 (Excoffier et al. 2013). Because the hybridization event most likely occurred prior to further lineage diversification (see *Results*) and to increase sample size per terminal and the number of retained SNPs, we pooled all genotyped populations into one of the three demes considered (*C. binotatus*, *C. saulcyi*, and the putative hybrid taxon *C. s. algoaldensis*) according to phylogenomic inferences (see *Results*; e.g., Eaton et al. 2015).

We calculated a folded joint SFS using the *easySFS.py* script (I. Overcast, https://github.com/isaacovercast/easySFS). We considered a single SNP per locus to avoid the effects of linkage disequilibrium and downsampled each population group (deme) to 50% of individuals to remove all missing data for the calculation of the joint SFS, minimize errors with allele frequency estimates, and maximize the number of variable SNPs retained. The final SFS contained 1998 variable SNPs. Because we did not include invariable sites in the SFS, we used the “removeZeroSFS” option in fastsimcoal2 and fixed the effective population size for one of the demes (*C. binotatus*) to enable the estimation of other parameters in fastsimcoal2 (Excoffier et al. 2013; Papadopoulou and Knowles 2015). The effective population size fixed in the model was calculated from the level of nucleotide diversity (π) and estimates of mutation rate per site per generation (μ), because *N*_e_ = (π/4μ). Nucleotide diversity (π) was estimated from polymorphic and nonpolymorphic loci using dnasp version 6.12.03 (Rozas et al. 2017). We considered the mutation rate per site per generation of 2.8 × 10^−9^ estimated for *Drosophila melanogaster* (Keightley et al. 2014).

Each model was run 100 replicated times considering 100,000–250,000 simulations for the calculation of the composite likelihood, 10–40 expectation‐conditional maximization (ECM) cycles, and a stopping criterion of 0.001 (Excoffier et al. 2013). We used an information‐theoretic model selection approach based on the Akaike's information criterion (AIC) to determine the probability of each model given the observed data (Burnham and Anderson 2002; e.g., Thomé and Carsterns 2016). After the maximum likelihood was estimated for each model in every replicate, we calculated the AIC scores as detailed in Thomé and Carsterns (2016). AIC values for each model were rescaled (ΔAIC) calculating the difference between the AIC value of each model and the minimum AIC obtained among all competing models (i.e., the best model has ΔAIC = 0). Point estimates of the different demographic parameters for the best supported model were selected from the run with the highest maximum composite likelihood. Finally, we calculated confidence intervals (based on the percentile method; e.g., de Manuel et al. 2016) of parameter estimates from 100 parametric bootstrap replicates by simulating SFS from the maximum composite likelihood estimates and re‐estimating parameters each time (Excoffier et al. 2013).

### PHENOTYPIC VARIATION ANALYSES

To assess the effect of hypothetical genetic admixture between parental taxa on the phenotype of the putative hybrid lineage (*C. s. algoaldensis*), we took digital images of taxonomically relevant traits (left hind femur, left forewing, and pronotum; Defaut 2011; Noguerales et al. 2018a) from each of the 231 sampled individuals (Table S1) and analyzed them by means of both linear and geometric morphometric approaches. For the linear morphology approach, we focused on three ratio traits that have been already considered in previous taxonomic studies of the group (Defaut 2011; Noguerales et al. 2018a), namely: (i) forewing length relative to femur length (FWL/FL), (ii) forewing median area length relative to total forewing length (MAL/FWL), and (iii) prozone length relative to total pronotum length (PZ/PR). Regarding the geometric morphometric approach, we focused on forewing shape (e.g., Klingenberg et al. 2010; Noguerales et al. 2018a; Tonzo et al. 2019). Variation in forewing shape was analyzed in morphoj version 1.05d (Klingenberg 2011) considering 10 homologous landmarks that have been previously shown to be highly informative in describing geometric morphometric variation of this trait within the study group (Noguerales et al. 2016, 2018a). Briefly, we conducted a Procrustes fit separately for each sex, removed the allometry effect on trait shape, and summarized size‐corrected shape variation by means of Principal Component Analyses (PCA) (for more details, see Klingenberg 2011). Differences among subspecies and among the three main groups (*C. s. algoaldensis* and its putative parental taxa *C. binotatus* and *C. saulcyi*) for ratio traits were tested using one‐way ANOVAs in r. Likewise, differences among subspecies and the three main groups in forewing shape were assessed by calculating Mahalanobis distances (*D*) from a canonical variate analyses (CVA) and conducting 10,000 permutation tests to calculate statistical significance (Klingenberg 2011). All traits were analyzed separately for each sex due to the considerable sexual size dimorphism in Orthoptera (Hochkirch and Gröning 2008; García‐Navas et al. 2017a).

## Results

### GENOMIC DATA

Illumina sequencing provided a total of 211.01 M sequences reads, with an average 2.45 M sequence reads per individual (SD = 0.39 M) (Fig. S2). After the different filtering and assembly steps, each individual retained on average 2.15 M sequence reads (SD = 0.35 M) (Fig. S2). After discarding clusters with less than five reads (see Methods S2), mean depth per locus was 15.19 (SD = 1.82) across individuals. The final dataset contained 17,598 variable unlinked SNPs with an average 60% of missing data. All pairs of genotyped individuals had negative relatedness values (ranging from −3.83 to −0.31), which excludes the possibility that we had sampled close relatives (Manichaikul et al. 2010).

### GENETIC CLUSTERING ANALYSES

Genetic clustering analyses in faststructure supported *K* = 2 and *K* = 6 as the most likely number of genetic groups according to the metrics *K* ^*^_ø_^c^ and *K* ^*^_ɛ_, respectively. When considering *K* = 2, the different taxa were assigned to either *C. binotatus* or *C. saulcyi* in concordance with the prevailing taxonomy of the group. For *K* = 6, *C. saulcyi* split into four genetic clusters that corresponded to *C. s. algoaldensis*, *C. s. moralesi*, *C. s. daimei*, and *C. s. saulcyi* together with the subspecies *C. s. vicdessossi*, whereas *C. binotatus* divided into two well‐defined genetic clusters corresponding to the Maghrebian (*C. b. atlasi*) and European (*C. b. binotatus* and *C. b. armoricanus*) taxa (Fig. 2). Evaluation of alternative *K*‐values within the range of best‐supported clustering solutions (*K* = 2–6) confirmed that genomic variation is hierarchically organized and infraspecific entities (i.e., subspecies and populations) are nested into well‐defined genetic clusters corresponding to the different putative taxa. Inferences assuming an increasing number of genetic clusters (*K* > 6) showed no further genetic structure and consistently yielded “ghost clusters” (i.e., clusters with no population or individual assigned to them; Guillot et al. 2005). DAPC also identified *K* = 6 as the most‐supported number of genetic clusters according to the Bayesian Information Criterion (BIC; Fig. S3a), confirming the main results yielded by faststructure (Fig. S3b). None of the clustering solutions based on either faststructure or DAPC showed evidence of recent genetic admixture at species, subspecies, or population levels (ancestry >99.99%; Figs. 2 and S3b).

### PHYLOGENOMIC INFERENCE

**Figure 3:**
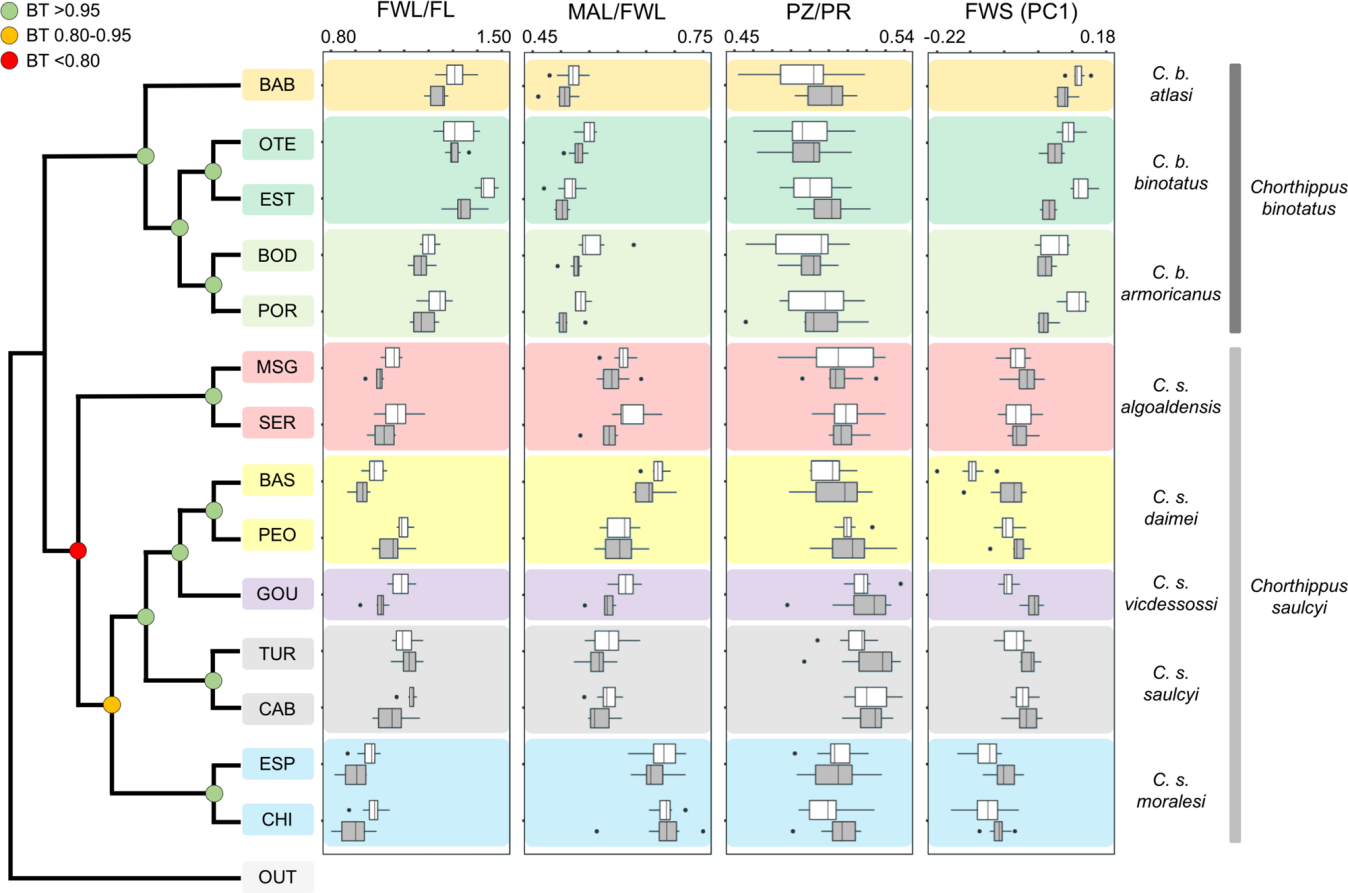
Species tree inferred with svdquartets showing the phylogenetic relationships among the different populations and taxa from the studied species complex. Bootstrapping values (BT) are indicated on the nodes using different colors as detailed in the legend. Boxplots on the right panels summarize phenotypic variation for each studied trait, including forewing length relative to femur length (FWL/FL), forewing median area length relative to forewing length (MAL/FWL), prozone length relative to pronotum length (PZ/PR), and forewing shape (FWS) variation based on the first principal component (PC1). Outliers are shown as black dots, and white and gray boxplots represent males and females, respectively. Population codes as in Table S1.

The species tree reconstructed in svdquartets revealed the existence of two major clades corresponding to the *C. binotatus* and *C. saulcyi* groups. In line with inferences from genetic clustering analyses, populations from the same putative subspecies grouped into well‐supported monophyletic subclades (Fig. 3). Although the relationships among subspecies from the *C. binotatus* group were well resolved (node support >99%), the phylogenetic position of *C. s. algoaldensis* was unclear and the node separating it from the rest of taxa within the *C. saulcyi* group was the one showing the lowest bootstrapping support (= 79%) in the whole tree (Fig. 3). The topology inferred by snapp was similar to that from svdquartets and also showed that the phylogenetic relationships within the *C. saulcyi* group were not well supported (Figs. 3 and S4). However, in this case the split concerning the focal taxon *C. s. algoaldensis* showed no evidence of topological uncertainty (Fig. S4). Replicate snapp runs considering different priors for the θ parameter converged on the same topology. Complementary svdquartets and snapp analyses excluding the putatively hybrid taxon *C. s. algoaldensis* yielded similar topologies and well‐supported basal nodes (Figs. S5 and S6; for a similar approach, see Rheindt et al. 2014).

### TESTING FOR INTROGRESSION

Results of *D*‐statistic tests revealed significant introgression involving *C. s. algoaldensis* and *C. binotatus* (*D*_s_ = 0.43 ± 0.06 SD; BABA = 65; ABBA = 103; *Z*‐score = 7.56; *P*‐value < 0.001; number of loci = 1245). Similar results were obtained when analyses were performed considering each taxon independently (i.e., subspecies within *C. binotatus* and *C. saulcyi*; Table S2). Significant introgression was also revealed between *C. b. atlasi* and any subspecies of *C. saulcyi*, as well as between *C. s. moralesi* and *C. binotatus* for most tested comparisons (Table S2).

### TESTING ALTERNATIVE DEMOGRAPHIC MODELS

**Figure 4:**
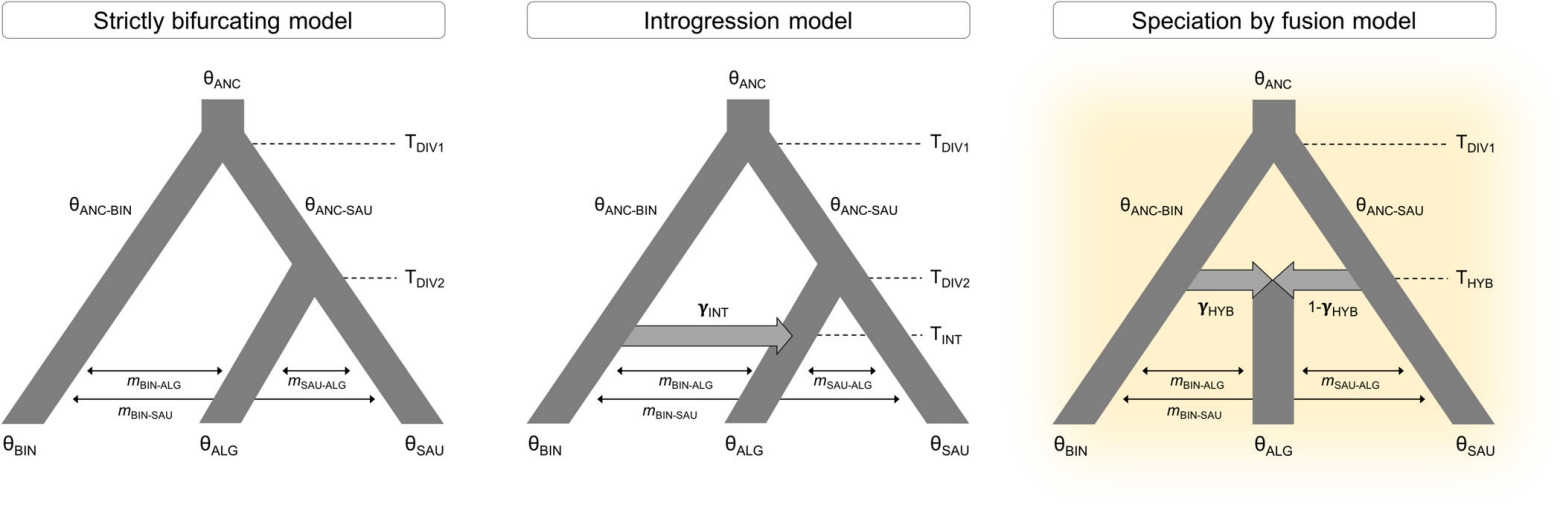
Alternative demographic scenarios tested using fastsimcoal2, including strictly bifurcating, introgression, and speciation by fusion models. Models were tested both considering and not considering postdivergence or post‐hybridization gene flow (see Table 1). Models assuming gene flow were built considering ancestral, contemporary, or both ancestral and contemporary migrations (see Fig. S1). Model parameters include ancestral (θ_anc_, θ_anc‐sau_, θ_anc‐bin_) and contemporary (θ_sau_, θ_alg_, θ_bin_) effective population sizes, timing of divergence (T_div1_, T_div2_), introgression (T_int_), and hybridization (T_hyb_), introgression (γ_int_) and hybridization (γ_hyb_) coefficients, and migration rates per generation (*m*). The best‐supported model is highlighted. Only models assuming contemporary gene flow are depicted. See Figure S1 for a detailed graphical description of all scenarios tested.

fastsimcoal2 analyses identified the speciation by fusion model incorporating contemporary gene flow as the most likely scenario (Table 1; Figs. 4 and S1). This result supported that *C. s. algoaldensis* originated from a hybridization event between *C. binotatus* and *C. saulcyi*. The introgression and strictly bifurcating models incorporating postdivergence gene flow were poorly supported (ΔAIC > 10; Table 1). Models that did not incorporate postdivergence gene flow or only considered ancestral gene flow were highly unlikely (ΔAIC > 38; Table 1). Considering that the studied taxa are univoltine (i.e., 1‐year generation time), fastsimcoal2 estimated that *C. binotatus* and *C. saulcyi* diverged from a common ancestor (T_div1_) about 2.1 Ma (95% confidence interval [CI]: 1.3–2.2 Ma; Table 2). The hybridization event (T_hyb_) that led to the formation of *C. s. algoaldensis* was estimated to have occurred about 1.4 Ma (95% CI: 1.0–1.6 Ma; Table 2). These analyses showed that about 24% of alleles (95% CI: 10–43%) of *C. s. algoaldensis* were inherited (γ_hyb_) from *C. binotatus* and post‐hybridization migration rates per generation (*m*) among all demes were consistently very low (<8 × 10^−8^; Table 2).

**Table 1:** Comparison of alternative models (see Figs. 4 and S1) tested using fastsimcoal2. The three main models (strictly bifurcating, introgression, and speciation by fusion) were built both considering and not considering postdivergence or post‐hybridization gene flow among demes. Models assuming gene flow were built considering ancestral, contemporary, or both ancestral and contemporary migrations. The best‐supported model is highlighted in bold

Model	Gene flow	lnL	*k*	AIC	ΔAIC	ω*_i_*
Strictly bifurcating	No	−3158.59	6	6329.19	121.53	0.00
Introgression	No	−3147.64	8	6311.28	103.62	0.00
Speciation by fusion	No	−3148.15	8	6312.31	104.65	0.00
Strictly bifurcating	Yes (ancestral)	−3137.26	7	6288.53	80.87	0.00
Introgression	Yes (ancestral)	−3113.94	9	6245.88	38.22	0.00
Speciation by fusion	Yes (ancestral)	−3126.03	9	6270.05	62.40	0.00
Strictly bifurcating	Yes (full migration)	−3101.75	10	6223.49	15.84	0.00
Introgression	Yes (full migration)	−3097.88	12	6219.77	12.11	0.00
Speciation by fusion	Yes (full migration)	−3093.97	12	6211.94	4.29	0.09
Strictly bifurcating	Yes (contemporary)	−3101.70	9	6221.39	13.74	0.00
Introgression	Yes (contemporary)	−3097.91	11	6217.82	10.17	0.01
**Speciation by fusion**	**Yes (contemporary)**	**−3092.83**	**11**	**6207.65**	**0.00**	**0.90**

lnL = maximum likelihood estimate of the model; *k* = number of parameters in the model; AIC = Akaike's information criterion value; ∆AIC = difference in AIC value from that of the strongest model; ω*_i_* = AIC weight.

**Table 2:** Parameters inferred from coalescent simulations with fastsimcoal2 under the best‐supported speciation by fusion model (see Fig. 4). For each parameter, we show its point estimate and lower and upper 95% confidence intervals. Model parameters include ancestral (θ_anc_, θ_anc‐sau_, θ_anc‐bin_) and contemporary (θ_sau_, θ_alg_) mutation‐scaled effective population sizes, timing of ancestral divergence (T_div1_) and hybridization (T_hyb_), admixture coefficient (γ_hyb_), and migration rates per generation (*m*) among demes. Note that the effective population size of *C. binotatus* (θ_bin_) is not presented because it was fixed in fastsimcoal2 analyses to enable the estimation of other parameters

Parameter	Point estimate	Lower bound	Upper bound
θ_anc_	40,956	33,427	527,869
θ_anc‐sau_	933,380	308,449	1,617,713
θ_anc‐bin_	9721	4915	272,882
θ_alg_	680,545	547,955	738,974
θ_sau_	2,834,490	2,329,980	3,079,558
T_hyb_	1,365,495	1,019,168	1,567,835
T_div1_	2,052,400	1,348,179	2,195,238
γ_hyb_	0.24	0.10	0.43
*m*_sau‐alg_	7.19 × 10^−8^	4.45 × 10^−8^	1.06 × 10^−7^
*m*_bin‐alg_	2.74 × 10^−8^	1.18 × 10^−8^	4.05 × 10^−8^
*m*_bin‐sau_	5.74 × 10^−8^	4.08 × 10^−8^	7.09 × 10^−8^

### PHENOTYPIC VARIATION ANALYSES

Linear morphological analyses showed significant differences among the main groups (*C. binotatus*, *C. saulcyi*, and *C. s. algoaldensis*) and subspecies for the three ratio traits in both sexes (one‐way ANOVAs; all *P*‐values < 0.001). Post hoc Tukey's tests at the group level revealed that only comparisons between *C. binotatus* and any of the other two groups (*C. saulcyi* and *C. s. algoaldensis*) showed significant differences (*P*‐values < 0.05) for any trait and sex (Table S3; Fig. 3). Post hoc tests at subspecies level showed that *C. s. algoaldensis* was significantly different from any taxon within *C. binotatus* for the forewing‐derived ratio traits (FWL/FL and MAL/FWL) in both sexes (all *P*‐values < 0.05; Table S4; Fig. 3). Within *C. saulcyi*, we also found significant differences among taxa for these two ratio traits in both sexes, particularly in comparisons involving *C. s. algoaldensis* and those subspecies that are particularly short winged (*C. s. daimei* and *C. s. moralesi*) (Table S4; Fig. 3). The pronotum‐derived ratio trait (PZ/PR) only showed significant differences in post hoc tests for a few comparisons, mainly when subspecies *C. b. atlasi* and *C. s. saulcyi* were involved (Table S4).

Morphometric geometric analyses on forewing shape showed that *C. s. algoaldensis* clustered within the rest of the *C. saulcyi* group and that the overlapping between *C. binotatus* and *C. saulcyi* groups was low (Figs. 3 and S7). Although Mahalanobis distances (*D*) were significantly different between all groups and subspecies for both sexes (Tables S5 and S6), greater differences were found between *C. binotatus* and *C. s. algoaldensis* than when this taxon was compared to *C. saulcyi* (Tables S5 and S6).

## Discussion

We present evidence for hybridization to be a key component in the diversification of a species complex of Gomphocerinae grasshoppers. Phylogenetic tests and inferences from coalescent‐based demographic simulations supported the hybrid origin of the narrowly distributed taxon *C. s. algoaldensis*, shedding light on its uncertain taxonomic position and providing a potential explanation for its dual host‐plant feeding regime. Although we cannot categorically conclude that isolating mechanisms (i.e., speciation itself) were triggered by hybridization, our study offers clues for alternative scenarios of hybrid speciation complementing more conservative definitions of this phenomenon proposed in previous literature (McCarthy et al. 1995; Buerkle et al. 2000; Gross and Rieseberg 2005). Below, we discuss the underlying biogeographic scenario and ecological factors that may have promoted the formation of *C. s. algoaldensis* and its persistence as an independently evolving lineage of hybrid origin.

### HYBRID SPECIATION IN *Chorthippus* GRASSHOPPERS?

It has been estimated that the genome of at least 10% of species of animals has been sculpted by episodes of interspecific gene flow (Mallet 2005, 2007). Our study adds to the accumulating evidence on this phenomenon by demonstrating that the grasshopper *C. s. algoaldensis* represents a relatively ancient lineage of hybrid origin and also revealing introgression signatures across other lineages of the species complex (Table S2). Rather than an unusual finding, these results reinforce the notion of hybridization and introgression as a common phenomenon in Gomphocerinae grasshoppers reported in recent literature (e.g., Rohde et al. 2015; Nolen et al. 2020; Tonzo et al. 2020). In line with inferences from phylogenetic tests (Table S2), coalescent‐based demographic analyses strongly supported a speciation by fusion model for *C. s. algoaldensis* (Table 1). This confirms the role of genetic admixture in shaping the evolutionary history of the focal taxon while simultaneously offering an explanation for its uncertain phylogenetic placement and conflicting gene tree topologies in the complex (Fig. 3; Noguerales et al. 2018a). The fact that the genomic signatures of hybridization were revealed by phylogenetic tests involving each extant parental lineage (i.e., the different subspecies from each parental taxon; Table S2) indicates that the fusion event leading to the formation of *C. s. algoaldensis* was ancient and predated the diversification of *C. binotatus* and *C. saulcyi* into their respective infraspecific lineages (e.g., Ortego and Knowles 2021). In contrast to previous studies documenting gene flow across species boundaries in other grasshoppers (e.g., Orr et al. 1994; Bridle et al. 2002; Nolen et al. 2020; Tonzo et al. 2020; Ortego and Knowles 2021), the recombinant genome of *C. s. algoaldensis* is compatible with hybrid speciation rather than with a scenario of bifurcating divergence followed by a pulse of gene flow (i.e., introgressive hybridization; Table 1). Thus, we argue that this taxon has a hybrid origin and might represent a putative case of homoploid hybrid speciation, as proposed for a handful of organisms including butterflies (Gompert et al. 2006), fruitflies (Schwarz et al. 2005), birds (Barrera‐Guzman et al. 2018), and fishes (Keller et al. 2013).

In the last decade, the growing literature documenting presumably homoploid hybrid species has been accompanied by research efforts for outlining the criteria that should be satisfied to support this speciation mode, namely: (i) genomes exhibit signatures of hybridization (*criterion 1*), (ii) hybrids are reproductively isolated from parental forms (*criterion 2*), and (iii) isolating mechanisms were triggered by hybridization (*criterion 3*) (Abbott et al. 2013; Schumer et al. 2014; but see Nieto‐Feliner et al. 2017). Our genomic data clearly demonstrate that *C. s. algoaldensis* has a hybrid origin (*criterion 1*) and several lines of indirect evidence suggest that contemporary populations of *C. s. algoaldensis* are reproductively isolated from the rest of taxa of the group (*criterion 2*). Even though its adjacent distribution with other taxa within the complex (e.g., *C. s. daimei* and *C. b. armoricanus*; with nearest populations <130 km apart; Fig. 2; Noguerales et al. 2018a) and, thus, likely opportunities for secondary contact resulting from range expansions promoted by the marked climatic oscillations that followed the speciation by fusion event (about 1.4 Ma; Table 2), we found no evidence for ongoing hybridization or recent genetic admixture between *C. s. algoaldensis* and parental taxa. In this line, estimated migration rates per generation between *C. s. algoaldensis* and parental taxa were extremely low (<8 × 10^−8^; Table 2) and clustering analyses revealed no signatures of genetic admixture among lineages (individual‐based cluster memberships >0.999%; Figs. 2 and S3b), indicating that extant populations are at genotypic equilibrium (Lawson et al. 2018). This supports the genetic cohesiveness and distinctiveness of *C. s. algoaldensis* and its long‐term persistence as an independently evolving lineage (Barton and Hewitt 1985; e.g., Gompert et al. 2006, Gompert et al. 2014).

Despite different lines of evidence that satisfy or lend support to the two first criteria for homoploid hybrid speciation, we cannot determine whether reproductive isolation evolved as a direct consequence of hybridization (*criterion 3*; Schumer et al. 2014). Although this criterion is nearly impossible to demonstrate in ancient events leading to hybrid speciation, we hypothesize a scenario where coupled shifts in display traits and mating preferences resulted from hybridization could have played a relevant role in the reproductive isolation between *C. s. algoaldensis* and parental taxa (Rosentahl 2013). Gomphocerinae grasshoppers use acoustic signals for species recognition and mate choice (Nattier et al. 2011; Song et al. 2020), a behavior that has been extensively documented to be involved in reproductive isolation (Perdeck 1958; Bridle and Butlin 2002). Males of *C. s. algoaldensis* exhibit a slightly distinct courtship song relative to the rest of taxa within the group (Defaut 2011), raising the question of whether these differences emerged directly through hybridization and were instrumental as a prezygotic isolating barrier during the early stages of the nascent hybrid species. Although it is also possible that song differences in *C. s. algoaldensis* evolved through mechanisms unrelated to hybridization (e.g., as a by‐product of genetic drift due to long‐term geographical isolation), the fact that the remaining taxa of the group produce very similar calling songs suggests that this alternative hypothesis is less plausible (Defaut 2011). The distinct courtship behavior of *C. s. algoaldensis* would also be in concordance with field and experimental studies revealing that male hybrids of Gomphocerinae grasshoppers exhibit novel, intermediate, or even more elaborated calling songs on which sexual section can act (Perdeck 1958; Vedenina and von Helversen 2003; reviewed in Mayer et al. 2010). As suggested in other putative hybrid species (e.g., Naisbit et al. 2001; Mavárez et al. 2006; Melo et al. 2009; Lamichhaney et al. 2018), assortative mating could have favored the rapid establishment of reproductive barriers with parental taxa and provided the behavioral context for the hybrid lineage to progress toward speciation (Rosenthal 2013; Lamichhaney et al. 2018; see also Vedenina and von Helversen 2003).

### PHENOTYPIC OUTCOMES OF HYBRIDIZATION

Demographic modeling estimated that the genome of *C. s. algoaldensis* presents a high level of admixed ancestry, which provides a mechanistic explanation for the phenotypic and ecological attributes of this taxon. The asymmetric genetic contribution from each parental lineage (about 24% and 76% of gene copies originated from *C. binotatus* and *C. saulcyi*, respectively; Table 2) is congruent with the fact that *C. s. algoaldensis* tends to exhibit a greater morphological affinity with taxa belonging to its putative species *C. saulcyi* than with lineages of *C. binotatus* (Figs. 2, 3, and S7). This intermediate pattern, as opposed to transgressive phenotypes outside the parental morphometric space, is compatible with a scenario of ancient hybridization involving recently diverged taxa with marked morphological similarities. As predicted by the theory, transgressive hybrid phenotypes are more prone to emerge with increasing parental genetic and phenotypic divergence (Rieseberg et al. 1999; Stelkens et al. 2009). Our results suggest that the dual feeding regime of *C. s. algoaldensis* could be a consequence of its recombinant genome, providing ground evidence for further investigating the potential role of hybridization on trophic niche expansion. Although we cannot discard that the dual feeding regime of *C. s. algoaldensis* is the result of post‐speciation selection and adaptation, some lines of evidence suggest that this alternative hypothesis is less plausible. Given that all *Chorthippus* species are graminivorous (Gangwere and Morales‐Agacino 1973; Gardiner and Hill 2004), it has been suggested a feeding regime based on scrub legumes represents the derived state (Picaud et al. 2002, 2003). Considering that feeding‐related traits are likely polygenic, with an underlying complex genetic architecture including genes involved in chemosensory functions, plant recognition, and detoxifying and metabolic pathways (Simon et al. 2015), we argue that the most parsimonious explanation for the dual feeding strategy in *C. s. algoaldensis* is that this trait is an outcome of hybridization rather than the result of a partial evolutionary reversal to the ancestral graminivorous state.

### A BIOGEOGRAPHIC SCENARIO FOR PLEISTOCENE HYBRID SPECIATION

Pleistocene climatic fluctuations in temperate regions have been hypothesized to increase opportunities for divergence in isolation through distributional shifts and range fragmentation (“species pump” hypothesis; Knowles 2001; e.g., Papadopoulou and Knowles 2015; Ortego and Knowles 2021). Alternatively, it has been proposed that such climatic dynamics could have also prevented speciation by eroding incipient divergences as a result of secondary contact and lineage fusion linked to range expansions during favorable periods (“melting pot” hypothesis; Klicka and Zink 1997; e.g., Ebdon et al. 2021). The two phenomena are not mutually exclusive and its relative importance is expected to be context dependent, with cyclical events of lineage fusion (i.e., homoploid lineage formation) and divergence depending on the species’ ecological attributes and the environmental conditions prevailing at each time period (Qiao et al. 2018; Maier et al. 2019; Ortego and Knowles 2021). According to our divergence time estimates, the clades *C. binotatus* and *C. saulcyi* split during the Gelasian (about 2.1 Ma; Table 2), which falls within the crown age estimated for the species group *Chorthippus binotatus* based on mtDNA data (about 1.5–3.0 Ma; García‐Navas et al. 2017b). As hypothesized for most Gomphocerinae grasshoppers (Mayer et al. 2010; Noguerales et al. 2018a; Ortego et al. 2021), the early diversification of the species complex would be compatible with a scenario of geographical isolation promoted by range fragmentation since the onset of the Pleistocene (Noguerales et al. 2018a; Tonzo and Ortego 2021). Given the contrasting feeding strategies exhibited by the two main clades, it would be also plausible that adaptive divergence linked to the usage of differing host plants contributed to the early diversification of the group, as documented for other Orthoptera (Apple et al. 2010; Grace et al. 2010). Rather than mutually exclusive, both ecologically driven divergent selection and allopatric divergence processes could have synergistically contributed to the radiation of *C. binotatus* and *C. saulcyi* clades (Hoskin et al. 2005). Given that the formation of new taxa is generally understood as a protracted process (Rosindell et al. 2010), the time elapsed between the onset of divergence and the identified hybridization event (Calabrian age, about 1.4 Ma; Table 2) that gave rise to the formation of *C. s. algoaldensis* could be interpreted as an estimate of the pace of speciation in this group (Dynessius and Jansson 2014; Sukumaran et al. 2021). Our coalescent‐based estimates indicate that reproductive isolation accumulated since the ancestral split was not enough to prevent interbreeding during at least about 0.8 Ma (Table 2; see also Hewitt 1999, 2011). This finding would agree with previous studies on insects, for which the minimum time for total hybrid inviability has been estimated in 2–4 Ma (Coyne and Orr 1997; Presgraves 2002). This would support the notion that ancestral diversification likely occurred in geographical isolation, in line with expectations from allopatric speciation models predicting that the completion of reproductive isolation is a more lengthy process when primarily driven by genetic drift (Coyne and Orr 1989).

The magnitude and duration of glacial‐interglacial pulses increased during the last stages of the Pleistocene (Head and Gibbard 2005; Hewitt 2011), which led to severe vegetation shifts and repeated phases of reduction in forest extension in favor of grasslands and shrub‐like habitats throughout Europe (Donders et al. 2021). Thereby, it is plausible that during this period *C. binotatus* and *C. saulcyi* experienced marked distributional shifts and some of their populations came into secondary contact and hybridized, resulting in the establishment of a hybrid population that rapidly underwent geographic isolation. As expected for hybrid swarms in which population sizes of initial parental demes can be different, the genomic contribution of each parental to the recombinant genome of *C. s. algoaldensis* was uneven (Nolte and Tautz 2010). Our suggested Pleistocene hybrid speciation model would be in concordance with the prediction that temporal changes in habitat distribution and structure increase opportunities of hybridization between formerly isolated lineages (Anderson 1948; Singhal et al. 2021). In situ persistence of the nascent hybrid species and its long‐lasting geographical isolation from parental lineages could be facilitated by the complex topography characterizing the Central Massif, a region that has been already documented to be an extra‐Mediterranean Pleistocene refugia for temperate species (Kropf et al. 2012; Schmitt and Varga 2012; Ursenbacher et al. 2015). Beyond geographic isolation in a topographically complex climate refugium, it is also possible that trophic niche expansion may have conferred an adaptive value to the hybrid lineage, particularly under the highly dynamic environmental conditions prevailing during the Pleistocene that might have resulted in spatial mismatches between the grasshopper climatic niche and host‐plant distributions (Noguerales et al. 2018b).

## Conclusions

We propose a hybrid speciation model where ancient admixture and allopatric isolation in climate refugia can provide a suitable context for hybrid lineages to isolate from parental populations and persist through evolutionary time (James and Abbott 2005; Duenez‐Guzman et al. 2009). This two‐stage scenario—rapid fusion of parental lineages and isolation of the hybrid swarm—emphasizes the potential importance of Pleistocene‐driven demographic dynamics to the formation of homoploid hybrid species. This hypothesis does not neglect the need of evaluating hybridization‐derived reproductive isolation (Schumer et al. 2014), but it intends to offer alternative pathways for understanding ancient events of homoploid hybrid speciation in which demonstrating that hybridization triggered reproductive isolation with parental lineages is challenging or virtually impossible (Nieto‐Feliner et al. 2017; Edelman and Mallet 2021). Collectively, our study provides insights on how the interplay between extended speciation duration and climate‐mediated increasing opportunities for gene flow may eventually promote hybrid speciation (Dynessius and Jansson 2014). As opposed to the classic view of melting pots preventing diversification (Klicka and Zink 1997; Ebdon et al. 2021), our results offer more nuanced insights into Pleistocene speciation by highlighting how a combination of allopatric divergence and subsequent hybridization can both contribute to diversification (Seehausen et al. 2014; Meier et al. 2017; Gillespie et al. 2020).

## AUTHOR CONTRIBUTIONS

VN and JO conceived and designed the study and analyses. VN and JO collected the samples. VN analyzed the data and wrote the manuscript with inputs from JO.

## DATA ARCHIVING

Raw Illumina reads have been deposited at the NCBI Sequence Read Archive (SRA) under BioProject PRJNA702631. Input files for all analyses are available for download from Dryad (https://doi.org/10.5061/dryad.fxpnvx0v1).

## CONFLICT OF INTEREST

The authors declare no conflict of interest.

Associate Editor: R. Unckless

Handling Editor: T. Chapman

## Supporting information


**Table S1**. Geographical location and taxonomic information of the sampled populations.
**Table S2**. Analyses of introgression using *D*‐statistic tests.
**Table S3**. Differences in morphological traits between the focal taxon *Chorthippus saulcyi algoaldensis and Chorthippus binotatus and Chorthippus saulcyi*.
**Table S4**. Differences in morphological traits between the different subspecies of the studied species complex.
**Table S5**. Mahalanobis distances between the focal taxon *Chorthippus saulcyi algoaldensis* and *Chorthippus binotatus and Chorthippus saulcyi* obtained for forewing shape.
**Table S6**. Mahalanobis distances between the different subspecies of the studied species complex obtained for forewing shape.
**Figure S1**. Alternative demographic scenarios tested using fastsimcoal2.
**Figure S2**. Number of reads per individual before and after different quality filtering steps.
**Figure S3**. Results of the Discriminant Analysis of Principal Components (DAPC).
**Figure S4**. Species tree for the studied species complex as inferred by snapp.
**Figure S5**. Species tree as inferred by svdquartets excluding *Chorthippus saulcyi algoaldensis*.
**Figure S6**. Species tree as inferred by snapp excluding *Chorthippus saulcyi algoaldensis*.
**Figure S7**. Principal Component Analyses (PCA) of forewing shape for the different populations and subspecies of the studied species complex.
**Methods S1**. Genomic library preparation.
**Methods S2**. Genomic data filtering and sequence assembly.
