# Supplementary material for: Genomic evidence of speciation by fusion in a recent radiation of grasshoppers
Source: Evolution. 2022 Jun 13;76(11):2618–33. doi: 10.1111/evo.14508 (PMC9796961; doi:10.1111/evo.14508)
Supplement: Supplementary file 1 — Table S1. Geographical location and taxonomic information of the sampled populations. Table S2. Analyses of introgression using D‐statistic tests. Table S3. Differences in morphological traits between the focal taxon Chorthippus saulcyi algoaldensis and Chorthippus binotatus and Chorthippus saulcyi. Table S4. Differences in morphological traits between the different subspecies of the studied species complex. Table S5. Mahalanobis distances between the focal taxon Chorthippus saulcyi algoaldensis and Chorthippus binotatus and Chorthippus saulcyi obtained for forewing shape. Table S6. Mahalanobis distances between the different subspecies of the studied species complex obtained for forewing shape. Figure S1. Alternative demographic scenarios tested using fastsimcoal2. Figure S2. Number of reads per individual before and after different quality filtering steps. Figure S3. Results of the Discriminant Analysis of Principal Components (DAPC). Figure S4. Species tree for the studied species complex as inferred by snapp. Figure S5. Species tree as inferred by svdquartets excluding Chorthippus saulcyi algoaldensis. Figure S6. Species tree as inferred by snapp excluding Chorthippus saulcyi algoaldensis. Figure S7. Principal Component Analyses (PCA) of forewing shape for the different populations and subspecies of the studied species complex. Methods S1. Genomic library preparation. Methods S2. Genomic data filtering and sequence assembly. [file EVO-76-2618-s001.pdf]

Supporting Information for

Genomic evidence of speciation by fusion in a recent radiation of grasshoppers

Víctor Noguerales and Joaquín Ortego

Journal: *Evolution*

Contents:

Supplementary tables

Table S1. Geographical location and taxonomic information of the sampled populations.
Table S2. Analyses of introgression using *D*-statistic tests.
Table S3. Differences in morphological traits between the focal taxon *Chorthippus saulcyi algoaldensis* and *Chorthippus binotatus* and *Chorthippus saulcyi*.
Table S4. Differences in morphological traits between the different subspecies of the studied species complex.
Table S5. Mahalanobis distances between the focal taxon *Chorthippus saulcyi algoaldensis* and *Chorthippus binotatus* and *Chorthippus saulcyi* obtained for forewing shape.
Table S6. Mahalanobis distances between the different subspecies of the studied species complex obtained for forewing shape.

Supplementary figures

Figure S1. Alternative demographic scenarios tested using FASTSIMCOAL2.
Figure S2. Number of reads per individual before and after different quality filtering steps.
Figure S3. Results of the discriminant analysis of principal components (DAPC).
Figure S4. Species tree for the studied species complex as inferred by SNAPP.
Figure S5. Species tree as inferred by SVDQUARTETS excluding *Chorthippus saulcyi algoaldensis*.
Figure S6. Species tree as inferred by SNAPP excluding *Chorthippus saulcyi algoaldensis*.
Figure S7. Principal component analyses (PCA) of forewing shape for the different populations and subspecies of the studied species complex.

Supplementary methods

Methods S1. Genomic library preparation.
Methods S2. Genomic data filtering and sequence assembly.

References

Supplementary tables

Table S1. Geographical location and taxonomic information of the sampled populations from the studied species complex. The number of analyzed individuals per population for genetic (n_{gen}) and morphological (n_{mor}) analyses are indicated.

Taxon	Taxon code	Population	Population code	Country	Latitude	Longitude	Elevation	n_{mor}	n_{gen}
Chorthippus binotatus atlasi	ATL	Bab Bou Idir	BAB	Morocco	34.07527	-4.12262	1509	10♂-10♀	7
Chorthippus binotatus binotatus	BIN	Campos de Otero	OTE	Spain	37.11013	-3.40512	2314	8♂-9♀	6
Chorthippus binotatus binotatus	BIN	Serra da Estrela	EST	Portugal	40.31733	-7.56841	1621	11♂-6♀	6
Chorthippus binotatus armoricanus	ARM	Bodélio	BOD	France	47.69921	-2.28028	64	8♂-8♀	7
Chorthippus binotatus armoricanus	ARM	Le Porge	POR	France	44.84311	-1.18749	7	8♂-6♀	6
Chorthippus saulcyi algoaldensis	ALG	Montselgues	MSG	France	44.54336	3.99774	955	8♂-8♀	5
Chorthippus saulcyi algoaldensis	ALG	Col de la Serreyrède	SER	France	44.10294	3.54140	1320	9♂-8♀	5
Chorthippus saulcyi daimei	DAI	La Bastide	BAS	France	43.74340	6.63963	1433	8♂-8♀	5
Chorthippus saulcyi daimei	DAI	Péone	PEO	France	44.11695	6.96018	1783	8♂-8♀	5
Chorthippus saulcyi vicdessossi	VIC	Goulier	GOU	France	42.73964	1.51423	1447	8♂-8♀	5
Chorthippus saulcyi saulcyi	SAU	Turó de l' Home	TUR	Spain	41.77240	2.44332	1622	8♂-8♀	5
Chorthippus saulcyi saulcyi	SAU	Serra de Cabrera	CAB	Spain	42.07893	2.40337	1291	8♂-8♀	5
Chorthippus saulcyi moralesi	MOR	Espés	ESP	Spain	42.44307	0.58389	1361	9♂-8♀	5
Chorthippus saulcyi moralesi	MOR	Chía	CHI	Spain	42.56833	0.41708	1900	8♂-9♀	5
Chorthippus biroi (outgroup)	-	Omalos	OUT	Greece	35.35373	23.91355	1100	-	6

Table S2. Analyses of introgression using *D*-statistic (ABBA/BABA) tests performed considering all possible subspecies combinations for *Chorthippus binotatus* and *Chorthippus saulcyi*, according to the phylogenetic inferences obtained in SVDQUARTETS and SNAPP. Comparisons involving the focal taxon *C. s. algoaldensis* are highlighted with grey background. The standard deviation (SD) of the *D*-statistic (*D*) was estimated for each comparison with 1000 bootstrap replicates. The number of informative sites (*n* loci) used in each comparison is also shown. *Chorthippus biroi* was used as an outgroup taxon. Taxon codes as in Table S1.

P_1	P_2	P_3	BABA sites	ABBA sites	*D* (± SD)	*Z*-score	*P*-value	*n* loci
SAU	ALG	ATL	43	85	0.33 ± 0.10	3.36	<0.001	525
MOR	ALG	ATL	46	82	0.28 ± 0.11	2.53	0.011	541
VIC	ALG	ATL	44	67	0.20 ± 0.14	1.40	0.161	471
DAI	ALG	ATL	45	85	0.31 ± 0.12	2.61	0.009	539
SAU	ALG	BIN	59	84	0.18 ± 0.09	1.97	0.048	1076
MOR	ALG	BIN	50	86	0.26 ± 0.08	3.28	<0.001	1062
VIC	ALG	BIN	36	60	0.26 ± 0.10	2.65	0.008	840
DAI	ALG	BIN	40	94	0.40 ± 0.08	4.83	<0.001	1023
SAU	ALG	ARM	48	127	0.45 ± 0.07	6.28	<0.001	1041
MOR	ALG	ARM	48	123	0.44 ± 0.08	5.85	<0.001	1025
VIC	ALG	ARM	41	123	0.50 ± 0.08	6.33	<0.001	988
DAI	ALG	ARM	34	83	0.41 ± 0.09	4.44	<0.001	816
MOR	SAU	ATL	18	12	-0.23 ± 0.14	1.66	0.096	340
DAI	SAU	ATL	11	16	0.18 ± 0.14	1.30	0.194	337
VIC	SAU	ATL	11	18	0.23 ± 0.22	1.03	0.303	320
DAI	MOR	ATL	9	21	0.42 ± 0.12	3.47	<0.001	343
VIC	MOR	ATL	10	22	0.39 ± 0.16	2.43	0.015	326
DAI	VIC	ATL	11	13	0.08 ± 0.19	0.39	0.697	328
MOR	SAU	BIN	13	11	-0.07 ± 0.12	0.60	0.549	495
DAI	SAU	BIN	9	13	0.16 ± 0.09	1.75	0.080	493
VIC	SAU	BIN	9	16	0.27 ± 0.20	1.33	0.184	463
DAI	MOR	BIN	9	15	0.26 ± 0.12	2.26	0.024	497
VIC	MOR	BIN	9	18	0.31 ± 0.17	1.82	0.069	468
DAI	VIC	BIN	9	11	0.11 ± 0.15	0.70	0.484	470
MOR	SAU	ARM	12	8	-0.21 ± 0.11	1.95	0.051	492
DAI	SAU	ARM	8	11	0.13 ± 0.11	1.19	0.234	490
VIC	SAU	ARM	8	14	0.26 ± 0.20	1.27	0.204	460
DAI	MOR	ARM	6	14	0.37 ± 0.09	4.12	<0.001	494
VIC	MOR	ARM	7	17	0.40 ± 0.15	2.75	0.006	465
DAI	VIC	ARM	8	9	0.05 ± 0.17	0.30	0.764	467
BIN	ATL	SAU	14	42	0.51 ± 0.11	4.60	<0.001	342
ARM	ATL	SAU	9	38	0.60 ± 0.09	6.49	<0.001	339
BIN	ARM	SAU	29	24	-0.09 ± 0.08	1.06	0.290	495
BIN	ATL	MOR	12	48	0.60 ± 0.09	6.73	<0.001	348

ARM	ATL	MOR	11	45	0.62 ± 0.08	7.36	<0.001	345
BIN	ARM	MOR	30	28	-0.04 ± 0.08	0.54	0.589	499
BIN	ATL	DAI	12	41	0.55 ± 0.11	5.01	<0.001	345
ARM	ATL	DAI	9	36	0.61 ± 0.10	6.05	<0.001	342
BIN	ARM	DAI	26	24	-0.05 ± 0.09	0.56	0.575	497
BIN	ATL	VIC	12	42	0.56 ± 0.11	5.18	<0.001	328
ARM	ATL	VIC	9	37	0.59 ± 0.10	5.72	<0.001	325
BIN	ARM	VIC	25	23	-0.03 ± 0.09	0.29	0.772	467

Table S3. Results of the analyses testing for differences in (i) forewing length relative to femur length (FWL/FL), (ii) forewing median area length relative to total forewing length (MAL/FWL), and (iii) prozone length relative to total pronotum length (PZ/PR) between the focal taxon *Chorthippus saulcyi algoaldensis*, *Chorthippus binotatus* and *Chorthippus saulcyi*, using post-hoc Tukey ´s tests. Adjusted *P*-values are reported, presented below the diagonal for males and above the diagonal for females, with values in bold indicating significant comparisons.

		C. binotatus	*C. s. algoaldensis*	*C. saulcyi*
FWL/FL	*C. binotatus*	-	**<0.001**	**<0.001**
	C. s. algoaldensis	**<0.001**	-	0.789
	C. saulcyi	**<0.001**	0.750	-
		C. binotatus	*C. s. algoaldensis*	*C. saulcyi*
MAL/FWL	*C. binotatus*	-	**<0.001**	**<0.001**
	C. s. algoaldensis	**<0.001**	-	**0.025**
	C. saulcyi	**<0.001**	0.290	-
		C. binotatus	*C. s. algoaldensis*	*C. saulcyi*
PZ/PR	*C. binotatus*	-	**0.014**	**<0.001**
	C. s. algoaldensis	**<0.001**	-	0.400
	C. saulcyi	**<0.001**	0.753	-

Table S4. Results of the analyses testing for differences in (i) forewing length relative to femur length (FWL/FL), (ii) forewing median area length relative to total forewing length (MAL/FWL), and (iii) prozone length relative to total pronotum length (PZ/PR) between the different subspecies of the studied species complex, using post-hoc Tukey ´s tests. Adjusted *P*-values are reported, presented below the diagonal for males and above the diagonal for females, with values in bold indicating significant comparisons. Subspecies codes as in Table S1.

			ATL	BIN	ARM	ALG	DAI	VIC	SAU	MOR
FWL/FL		ATL	-	**0.010**	0.094	**<0.001**	**<0.001**	**<0.001**	**<0.001**	**<0.001**
	C. binotatus	BIN	0.061	-	**<0.001**	**<0.001**	**<0.001**	**<0.001**	**<0.001**	**<0.001**
		ARM	**0.004**	**<0.001**	-	**<0.001**	**<0.001**	**<0.001**	**0.001**	**<0.001**
		ALG	**<0.001**	**<0.001**	**<0.001**	-	0.967	0.999	**0.002**	**<0.001**
		DAI	**<0.001**	**<0.001**	**<0.001**	0.914	-	0.999	**<0.001**	**<0.001**
	C. saulcyi	VIC	**<0.001**	**<0.001**	**<0.001**	0.967	0.448	-	**0.008**	**<0.001**
		SAU	**<0.001**	**<0.001**	**<0.001**	0.165	**0.005**	0.958	-	**<0.001**
		MOR	**<0.001**	**<0.001**	**<0.001**	**<0.001**	**0.011**	**<0.001**	**<0.001**	**-**
			ATL	BIN	ARM	ALG	DAI	VIC	SAU	MOR
MAL/FWL		ATL	-	0.960	0.972	**<0.001**	**<0.001**	**<0.001**	**<0.001**	**<0.001**
	C. binotatus	BIN	0.990	-	0.999	**<0.001**	**<0.001**	**<0.001**	**<0.001**	**<0.001**
		ARM	0.199	0.466	-	**<0.001**	**<0.001**	**<0.001**	**<0.001**	**<0.001**
		ALG	**<0.001**	**<0.001**	**<0.001**	-	**0.001**	0.999	0.479	**<0.001**
		DAI	**<0.001**	**<0.001**	**<0.001**	0.284	-	**0.003**	**<0.001**	**<0.001**
	C. saulcyi	VIC	**<0.001**	**<0.001**	**<0.001**	0.999	0.460	-	0.956	**<0.001**
		SAU	**<0.001**	**<0.001**	**0.007**	**0.025**	**<0.001**	0.200	-	**<0.001**
		MOR	**<0.001**	**<0.001**	**<0.001**	**<0.001**	**<0.001**	**<0.001**	**<0.001**	**-**
			ATL	BIN	ARM	ALG	DAI	VIC	SAU	MOR
PZ/PR		ATL	-	0.992	0.904	0.838	0.747	0.108	**0.001**	0.960
	C. binotatus	BIN	0.999	-	0.999	0.200	0.133	**0.006**	**<0.001**	0.403
		ARM	0.999	0.999	-	0.060	**0.036**	**0.001**	**<0.001**	0.149
		ALG	**0.022**	**0.013**	**0.035**	-	0.999	0.674	**0.049**	0.999
		DAI	**0.057**	**0.044**	0.098	0.999	-	0.769	0.080	0.998
	C. saulcyi	VIC	**<0.001**	**<0.001**	**<0.001**	0.574	0.403	-	0.991	0.429
		SAU	**<0.001**	**<0.001**	**<0.001**	0.233	0.121	0.999	-	**0.011**
		MOR	0.221	0.224	0.377	0.963	0.996	0.128	**0.016**	**-**

Table S5. Mahalanobis distances (*D*) between the focal taxon *Chorthippus saulcyi algoaldensis*, *Chorthippus binotatus* and *Chorthippus saulcyi* obtained through a Canonical Variates Analysis (CVA) for forewing shape. Values for males are presented below the diagonal and for females above the diagonal. Values in bold indicate significant Mahalanobis distances (*D*).

	C. binotatus	*C. s. algoaldensis*	*C. saulcyi*
C. binotatus	-	**4.372**	**4.557**
C. s. algoaldensis	**5.412**	-	**2.031**
C. saulcyi	**6.252**	**2.972**	-

Table S6. Mahalanobis distances (*D*) between the different subspecies of the studied species complex obtained through a Canonical Variates Analysis (CVA) for forewing shape. Values for males are presented below the diagonal and for females above the diagonal. Values in bold indicate significant Mahalanobis distances (*D*). Subspecies codes as in Table S1.

		ATL	BIN	ARM	ALG	DAI	VIC	SAU	MOR
C. binotatus	ATL	-	**3.813**	**4.358**	**6.431**	**7.866**	**5.825**	**5.133**	**9.841**
	BIN	**2.538**	-	**2.616**	**6.028**	**7.277**	**5.480**	**4.859**	**9.004**
	ARM	**2.521**	**2.227**	-	**5.148**	**6.149**	**4.580**	**4.401**	**7.994**
C. saulcyi	ALG	**7.880**	**6.716**	**7.072**	-	**2.886**	**3.708**	**3.034**	**4.431**
	DAI	**9.276**	**8.302**	**8.337**	**3.201**	-	**4.360**	**4.330**	**3.350**
	VIC	**7.382**	**6.700**	**6.849**	**3.956**	**3.110**	-	**2.580**	**6.060**
	SAU	**6.754**	**6.103**	**6.207**	**3.592**	**3.658**	1.807	-	**6.258**
	MOR	**11.796**	**10.587**	**10.665**	**5.318**	**3.974**	**6.054**	**6.331**	**-**

Supplementary figures

Figure S1. Alternative demographic scenarios tested using FASTSIMCOAL2. Models were tested both considering and not considering post-divergence or post-hybridization gene flow. Models assuming gene flow were built considering ancestral, contemporary or both ancestral and contemporary migrations. Model parameters include ancestral (θ_{ANC}, $\theta_{ANC\text{-}SAU}$, $\theta_{ANC\text{-}BIN}$) and contemporary (θ_{SAU}, θ_{ALG}, θ_{BIN}) effective population sizes, timing of divergence (T_{DIV1}, T_{DIV2}), introgression (T_{INT}) and hybridization (T_{HYB}), introgression (γ_{INT}) and hybridization (γ_{HYB}) coefficients, and migration rates per generation (*m*). The best-supported model is highlighted.

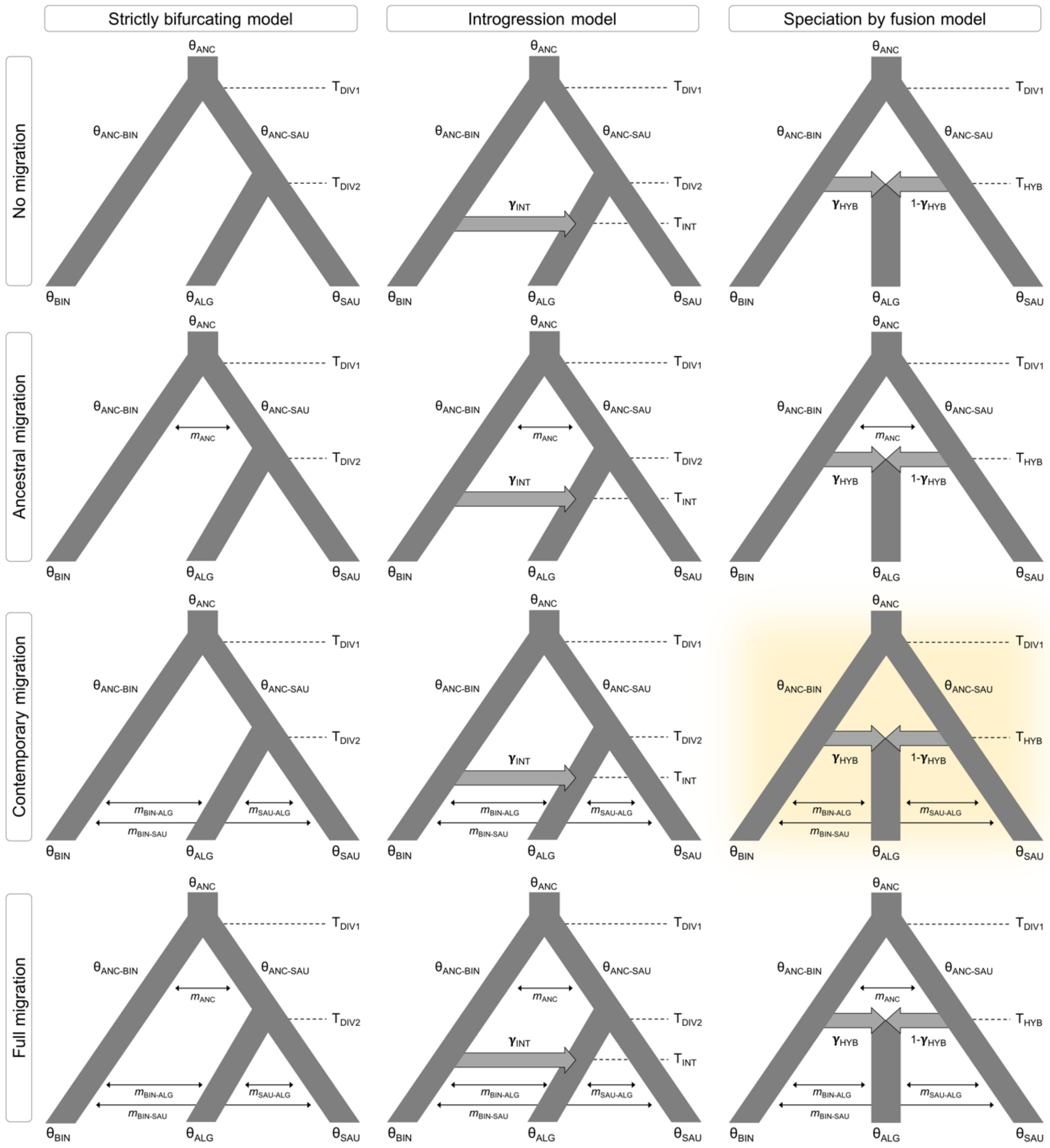

Figure S2. Number of reads per individual before and after different quality filtering steps by STACKS and PYRAD. The cumulative stacked bars represent the total number of raw reads obtained for each individual. Within each bar, the dark grey color represents the reads that were discarded by STACKS (*process_radtags*) due to low quality, adapter contamination or an ambiguous barcode. Medium grey color represents the reads that were subsequently discarded by PYRAD after filtering out reads that did not comply with the quality criteria (reads with >2 sites with a Phred quality score <20 were discarded). Finally, light grey color represents the total number of retained reads used to identify homologous loci during the subsequent steps performed in PYRAD. Grey horizontal line indicates the average number of reads across all individuals (*n* = 83). Population codes as in Table S1.

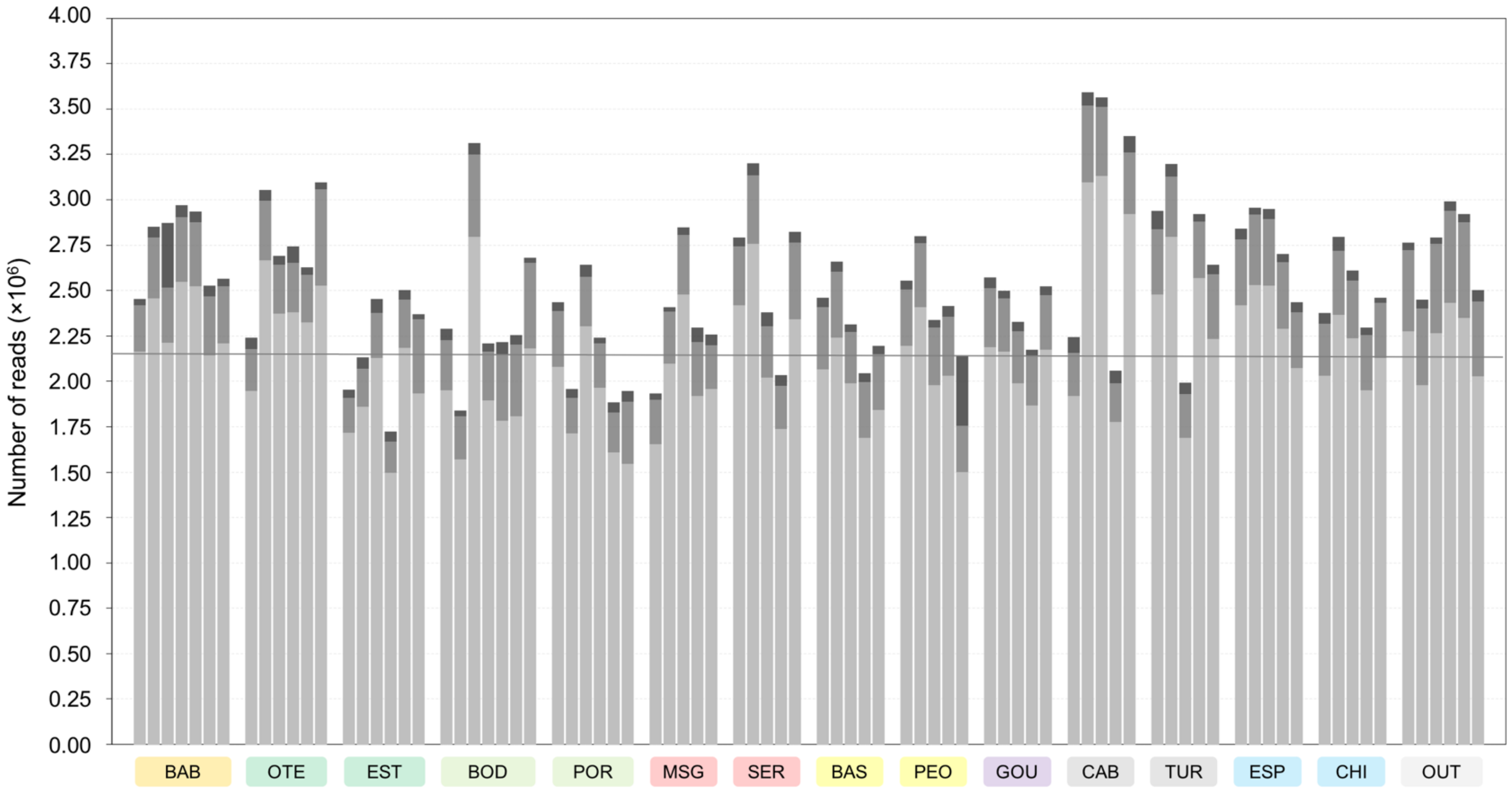

Figure S3. Results of the discriminant analysis of principal components (DAPC). Panel (a) represents the Bayesian Information Criterion (BIC) for increasing values of genetic clusters (*K*). Panel (b) shows the inferred genetic clustering from *K* = 3 to *K* = 6. Individuals are partitioned into *K* colored segments representing the probability of belonging to the cluster with that color. Thin vertical black lines separate different populations. Population codes as in Table S1.

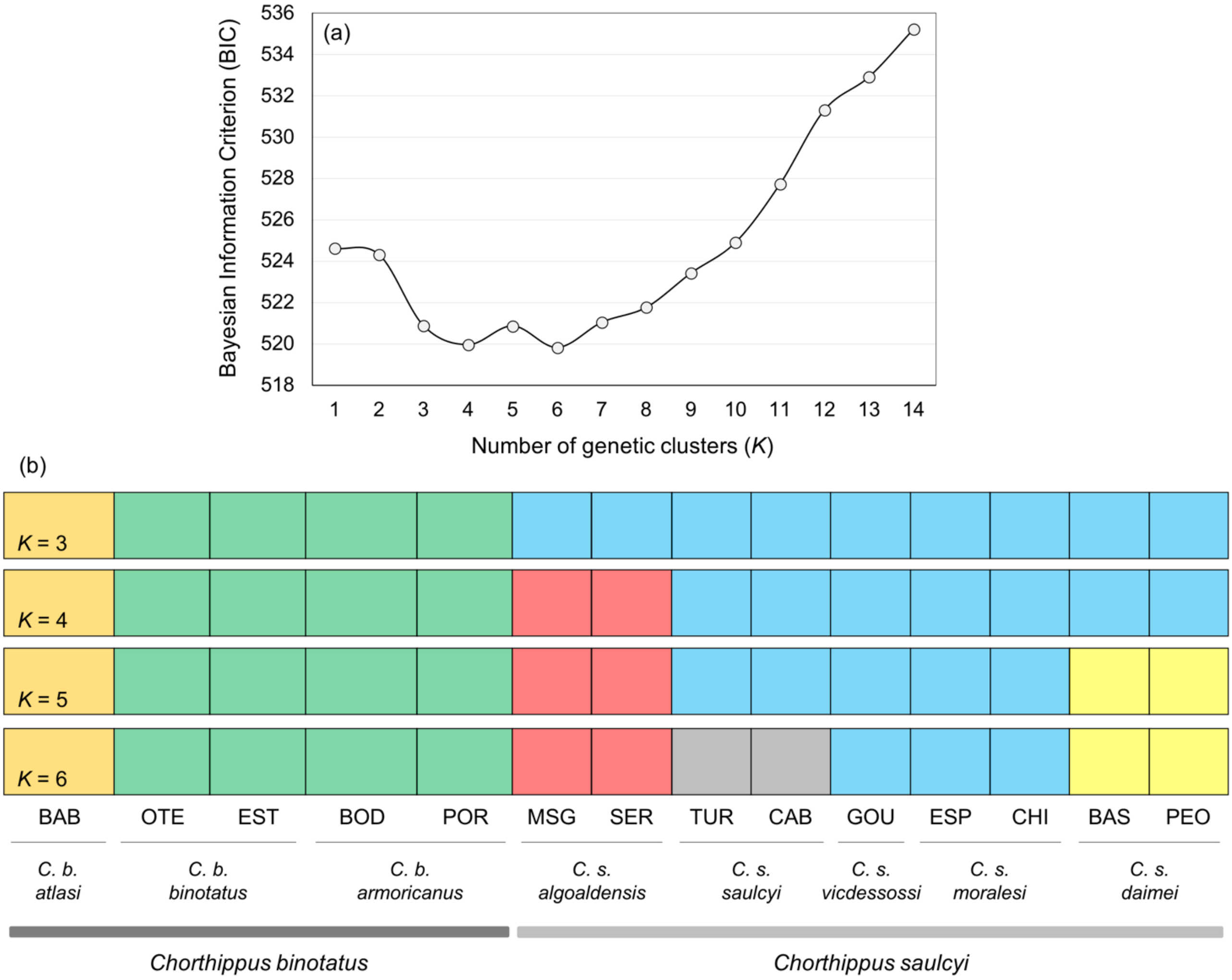

Figure S4. Species tree as inferred by SNAPP (based on 2926 bi-allelic unlinked SNPs) displaying the phylogenetic relationships among taxa from the studied species complex. Posterior probabilities for the most supported topology are indicated on the nodes. Taxon codes as in Table S1.

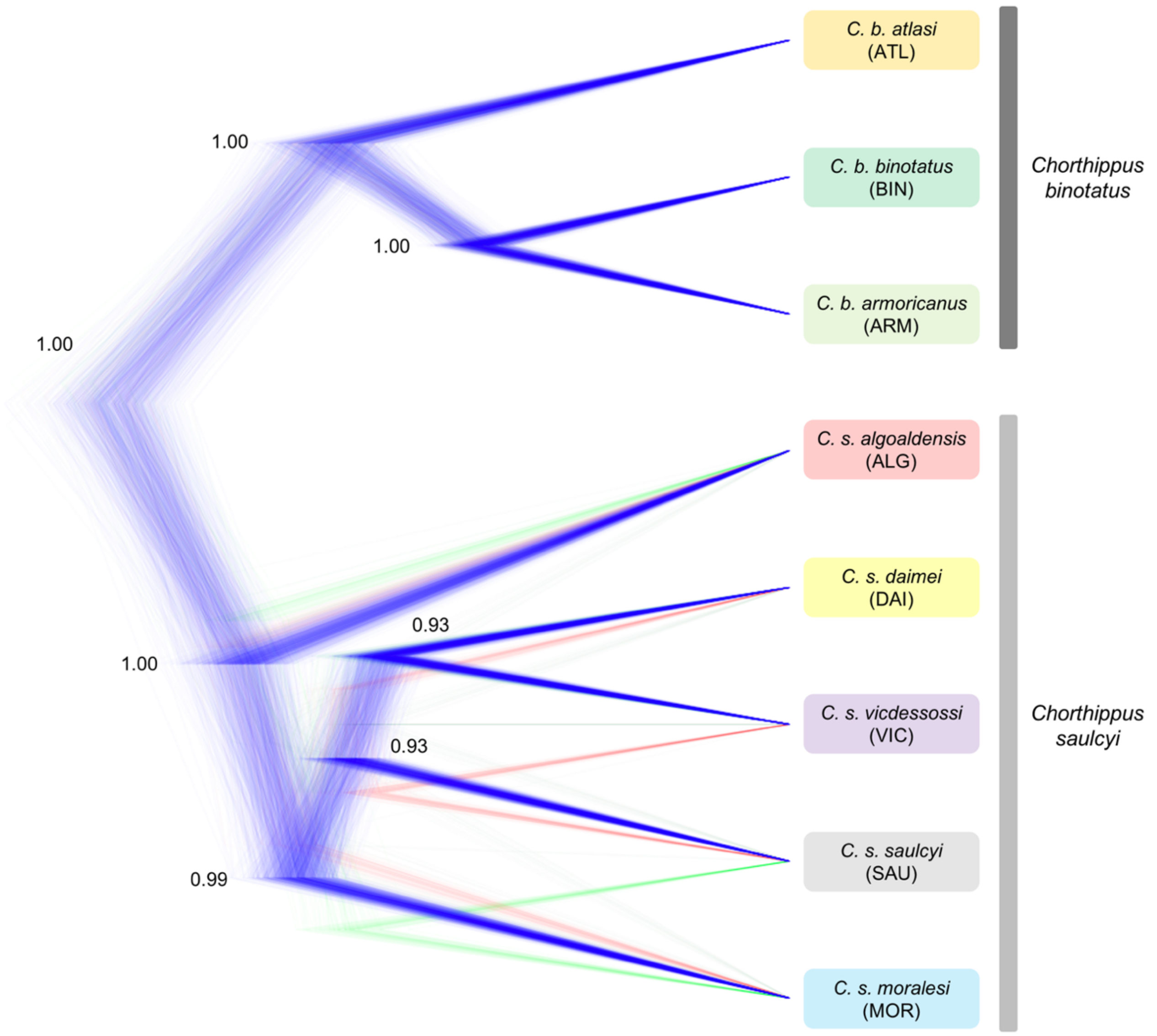

Figure S5. Species tree inferred with SVDQUARTETS showing the phylogenetic relationships among the different populations and taxa from the studied species complex, once the focal taxon *Chorthippus saulcyi algoaldensis* was excluded (see Fig. 3 and Fig. S4). Bootstrapping values (BT) are indicated on the nodes. Population codes as in Table S1.

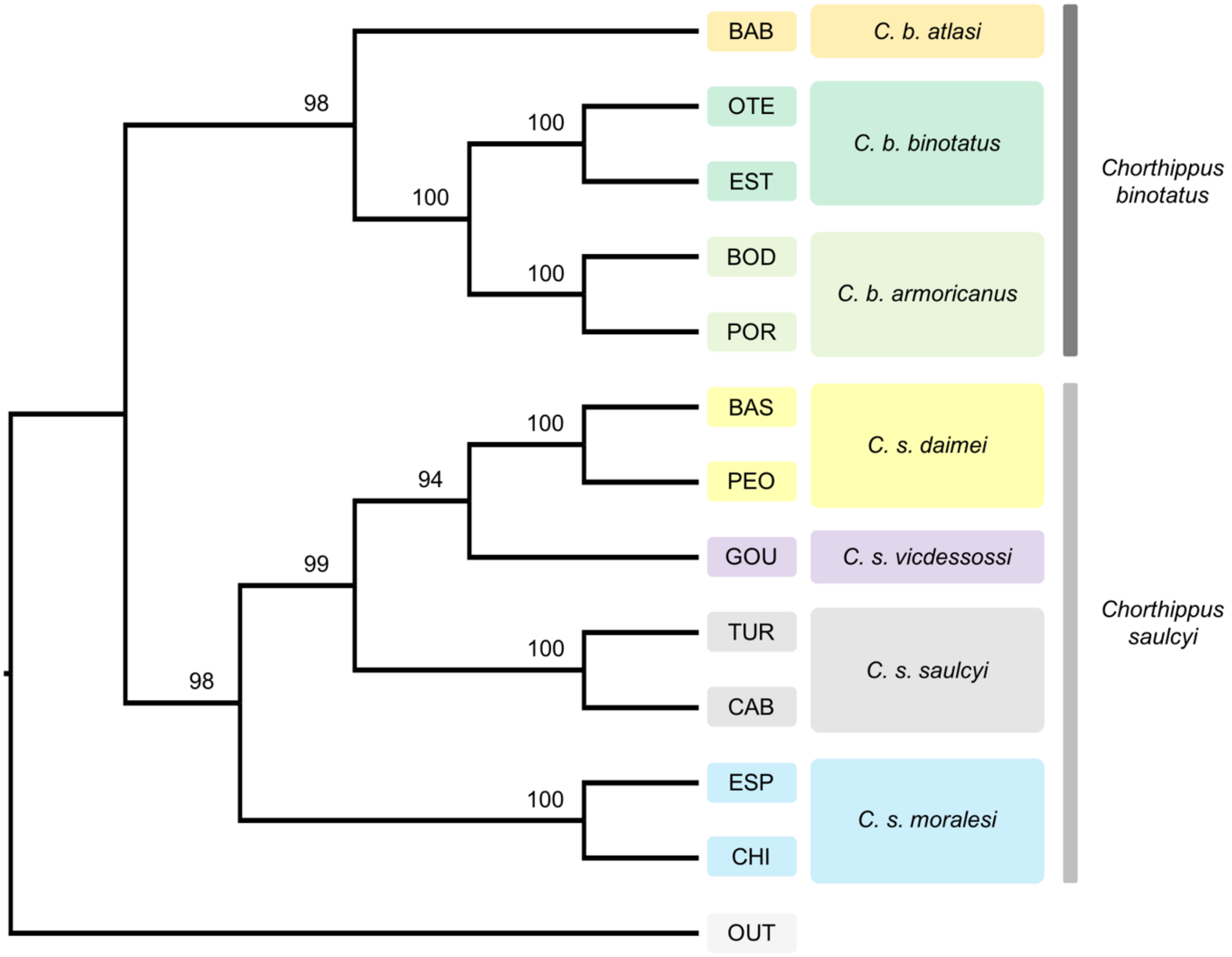

Figure S6. Species tree as inferred by SNAPP (based on 3,092 bi-allelic unlinked SNPs) displaying the phylogenetic relationships among taxa from the studied species complex, once the focal taxon *Chorthippus saulcyi algoaldensis* was excluded (see Fig. 3 and Fig. S4). Posterior probabilities for the most supported topology are indicated on the nodes. Taxon codes as in Table S1.

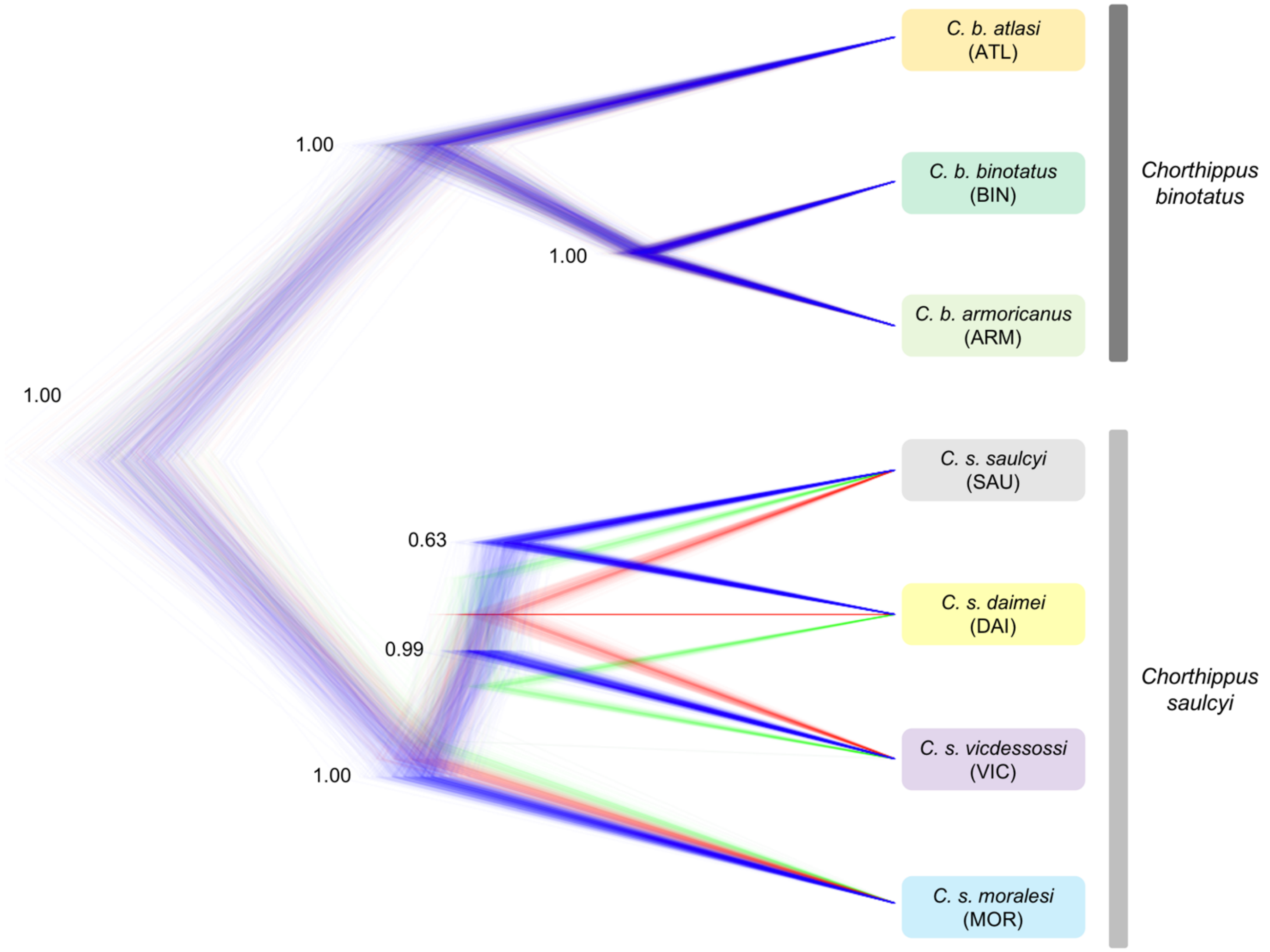

Figure S7. Forewing shape variation in males (top panel) and females (bottom panel) for the different subspecies and populations of the studied complex along the two first principal components (PCs). Population codes as in Table S1.

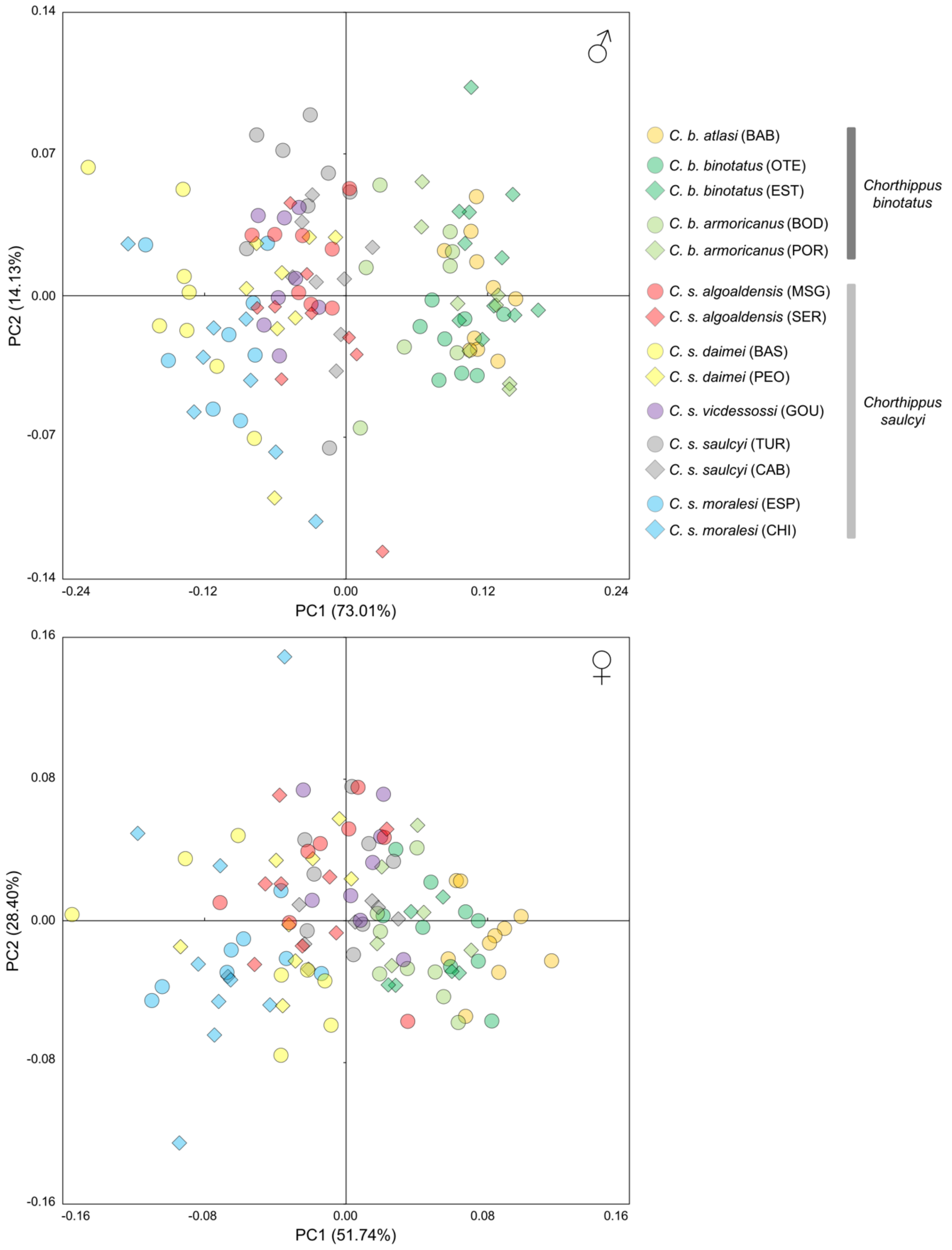

Supplementary methods

Methods S1. Genomic library preparation.

We used a salt extraction protocol to purify genomic DNA from a hind femur of each specimen (Aljanabi and Martinez 1997). Genomic DNA from each specimen was individually barcoded and processed into a genomic library using the double digestion restriction associated DNA sequencing (ddRADseq) procedure described in Peterson et al (2012) with some minor modifications as detailed in Lanier et al. (2015) and Massatti and Knowles (2016). Briefly, DNA was double-digested using *Eco*RI and *Mse*I restriction enzymes (New England Biolabs), followed by the ligation of Illumina adaptors and unique 7-base-pair barcodes. Ligation products were pooled, size-selected between 475 and 580 base pairs (bp) using a Pippin Prep (Sage Science) machine, and amplified by iProof™ High-Fidelity DNA Polymerase (BIO-RAD) with 12 cycles. Single-read 151 bp sequencing was performed on an Illumina HiSeq2500 platform at The Centre for Applied Genomics (Hospital for Sick Children, Toronto, Canada).

Methods S2. Genomic data filtering and sequence assembly.

Raw sequence reads were demultiplexed and quality-filtered using the *process_radtags* script within the STACKS v.1.35 pipeline (Catchen et al. 2013). Only reads with a Phred score >10 (using a sliding window of 15%), no adaptor contamination, and that had an unambiguous barcode and restriction cut site were retained. Then, we checked the quality of raw sequences in FASTQC v.0.11.5 (*http://www.bioinformatics.babraham.ac.uk/projects/fastqc/*) and trimmed them to 129 bp using SEQTK (Heng Li, *https://github.com/lh3/seqtk*) in order to remove low-quality reads near the 3´ ends. Reads retained after *process_radtags* were further quality-filtered using the program PYRAD v.3.0.66 (Eaton 2014) to convert base calls with a Phred score <20 into Ns and discard reads with >2 Ns (Fig. S1). Afterwards, we used PYRAD to cluster retained reads within- and across samples considering a clustering threshold of sequence similarity of 85% (W_{CLUST} = 0.85). Clusters with a minimum coverage depth less than 5 (*d* = 5) and loci containing one or more heterozygous sites across more than 15% of individuals (*maxSH* = p.15) and more than 20 polymorphic sites (*maxSNPs* = 20) were discarded (Eaton 2014; Hipp et al. 2014). In a final filtering step, we retained those loci that were present in at least 25% of the samples (minimum taxon coverage, *minCov* = 25%; Noguerales et al. 2018), which retained a total of 17,598 unlinked SNPs.

References

Aljanabi, S. M. and I. Martinez. 1997. Universal and rapid salt-extraction of high quality genomic DNA for PCR-based techniques. Nucleic Acids Res. 25:4692-4693.

Catchen, J., P. A. Hohenlohe, S. Bassham, A. Amores, and W. A. Cresko. 2013. STACKS: an analysis tool set for population genomics. Mol. Ecol. 22:3124-3140.

Eaton, D. A. R. 2014. PYRAD: assembly of de novo RADseq loci for phylogenetic analyses. Bioinformatics 30:1844-1849.

Hipp, A. L., D. A. R. Eaton, J. Cavender-Bares, E. Fitzek, R. Nipper, and P. S. Manos. 2014. A framework phylogeny of the American oak clade based on sequenced RAD data. PLoS One 9:e102272.

Lanier, H. C., R. Massatti, Q. He, L. E. Olson, and L. L. Knowles. 2015. Colonization from divergent ancestors: glaciation signatures on contemporary patterns of genomic variation in Collared Pikas (*Ochotona collaris*). Mol. Ecol. 24:3688-3705.

Massatti, R., and L. L. Knowles. 2016. Contrasting support for alternative models of genomic variation based on microhabitat preference: species-specific effects of climate change in alpine sedges. Mol. Ecol. 25:3974-3986.

Noguerales, V., P. J. Cordero, and J. Ortego. 2018. Integrating genomic and phenotypic data to evaluate alternative phylogenetic and species delimitation hypotheses in a recent evolutionary radiation of grasshoppers. Mol. Ecol. 27:1229-1244.

Peterson, B. K., J. N. Weber, E. H. Kay, H. S. Fisher, and H. E. Hoekstra. 2012. Double digest RADseq: an inexpensive method for *de novo* SNP discovery and genotyping in model and non-model species. PLoS One 7:e37135.
